# Search for *CP* violation in ${{{\textrm{D}}}^{{0}}} \rightarrow {{\textrm{K}} _{\text {S}}^{{0}}} {{\textrm{K}} _{\text {S}}^{{0}}}$ decays in proton–proton collisions at $\sqrt{s} = 13\,\text {Te}\hspace{-.08em}\text {V}$

**DOI:** 10.1140/epjc/s10052-024-13244-0

**Published:** 2024-12-06

**Authors:** CMS Collaboration, A. Hayrapetyan, A. Tumasyan, W. Adam, J. W. Andrejkovic, T. Bergauer, S. Chatterjee, K. Damanakis, M. Dragicevic, P. S. Hussain, M. Jeitler, N. Krammer, A. Li, D. Liko, I. Mikulec, J. Schieck, R. Schöfbeck, D. Schwarz, M. Sonawane, S. Templ, W. Waltenberger, C.-E. Wulz, M. R. Darwish, T. Janssen, T. Van Laer, P. Van Mechelen, N. Breugelmans, J. D’Hondt, S. Dansana, A. De Moor, M. Delcourt, F. Heyen, S. Lowette, I. Makarenko, D. Müller, S. Tavernier, M. Tytgat, G. P. Van Onsem, S. Van Putte, D. Vannerom, B. Bilin, B. Clerbaux, A. K. Das, G. De Lentdecker, H. Evard, L. Favart, P. Gianneios, J. Jaramillo, A. Khalilzadeh, F. A. Khan, K. Lee, M. Mahdavikhorrami, A. Malara, S. Paredes, M. A. Shahzad, L. Thomas, M. Vanden Bemden, C. Vander Velde, P. Vanlaer, M. De Coen, D. Dobur, G. Gokbulut, Y. Hong, J. Knolle, L. Lambrecht, D. Marckx, K. Mota Amarilo, A. Samalan, K. Skovpen, N. Van Den Bossche, J. van der Linden, L. Wezenbeek, A. Benecke, A. Bethani, G. Bruno, C. Caputo, J. De Favereau De Jeneret, C. Delaere, I. S. Donertas, A. Giammanco, A. O. Guzel, Sa. Jain, V. Lemaitre, J. Lidrych, P. Mastrapasqua, T. T. Tran, S. Wertz, G. A. Alves, M. Alves Gallo Pereira, E. Coelho, G. Correia Silva, C. Hensel, T. Menezes De Oliveira, C. Mora Herrera, A. Moraes, P. Rebello Teles, M. Soeiro, A. Vilela Pereira, W. L. Aldá Júnior, M. Barroso Ferreira Filho, H. Brandao Malbouisson, W. Carvalho, J. Chinellato, E. M. Da Costa, G. G. Da Silveira, D. De Jesus Damiao, S. Fonseca De Souza, R. Gomes De Souza, M. Macedo, J. Martins, L. Mundim, H. Nogima, J. P. Pinheiro, A. Santoro, A. Sznajder, M. Thiel, C. A. Bernardes, L. Calligaris, T. R. Fernandez Perez Tomei, E. M. Gregores, B. Lopes Da Costa, I. Maietto Silverio, P. G. Mercadante, S. F. Novaes, B. Orzari, Sandra S. Padula, A. Aleksandrov, G. Antchev, R. Hadjiiska, P. Iaydjiev, M. Misheva, M. Shopova, G. Sultanov, A. Dimitrov, L. Litov, B. Pavlov, P. Petkov, A. Petrov, E. Shumka, S. Keshri, S. Thakur, T. Cheng, T. Javaid, L. Yuan, Z. Hu, Z. Liang, J. Liu, K. Yi, G. M. Chen, H. S. Chen, M. Chen, F. Iemmi, C. H. Jiang, A. Kapoor, H. Liao, Z.-A. Liu, R. Sharma, J. N. Song, J. Tao, C. Wang, J. Wang, Z. Wang, H. Zhang, J. Zhao, A. Agapitos, Y. Ban, S. Deng, B. Guo, C. Jiang, A. Levin, C. Li, Q. Li, Y. Mao, S. Qian, S. J. Qian, X. Qin, X. Sun, D. Wang, H. Yang, L. Zhang, Y. Zhao, C. Zhou, S. Yang, Z. You, K. Jaffel, N. Lu, G. Bauer, B. Li, J. Zhang, X. Gao, Z. Lin, C. Lu, M. Xiao, C. Avila, D. A. Barbosa Trujillo, A. Cabrera, C. Florez, J. Fraga, J. A. Reyes Vega, F. Ramirez, C. Rendón, M. Rodriguez, A. A. Ruales Barbosa, J. D. Ruiz Alvarez, D. Giljanovic, N. Godinovic, D. Lelas, A. Sculac, M. Kovac, A. Petkovic, T. Sculac, P. Bargassa, V. Brigljevic, B. K. Chitroda, D. Ferencek, K. Jakovcic, S. Mishra, A. Starodumov, T. Susa, A. Attikis, K. Christoforou, A. Hadjiagapiou, C. Leonidou, J. Mousa, C. Nicolaou, L. Paizanos, F. Ptochos, P. A. Razis, H. Rykaczewski, H. Saka, A. Stepennov, M. Finger, M. Finger, A. Kveton, E. Carrera Jarrin, Y. Assran, B. El-mahdy, S. Elgammal, A. Lotfy, M. A. Mahmoud, K. Ehataht, M. Kadastik, T. Lange, S. Nandan, C. Nielsen, J. Pata, M. Raidal, L. Tani, C. Veelken, H. Kirschenmann, K. Osterberg, M. Voutilainen, S. Bharthuar, N. Bin Norjoharuddeen, E. Brücken, F. Garcia, P. Inkaew, K. T. S. Kallonen, T. Lampén, K. Lassila-Perini, S. Lehti, T. Lindén, L. Martikainen, M. Myllymäki, M. M. Rantanen, H. Siikonen, J. Tuominiemi, P. Luukka, H. Petrow, M. Besancon, F. Couderc, M. Dejardin, D. Denegri, J. L. Faure, F. Ferri, S. Ganjour, P. Gras, G. Hamel de Monchenault, M. Kumar, V. Lohezic, J. Malcles, F. Orlandi, L. Portales, A. Rosowsky, M. Ö. Sahin, A. Savoy-Navarro, P. Simkina, M. Titov, M. Tornago, F. Beaudette, G. Boldrini, P. Busson, A. Cappati, C. Charlot, M. Chiusi, F. Damas, O. Davignon, A. De Wit, I. T. Ehle, B. A. Fontana Santos Alves, S. Ghosh, A. Gilbert, R. Granier de Cassagnac, A. Hakimi, B. Harikrishnan, L. Kalipoliti, G. Liu, M. Nguyen, C. Ochando, R. Salerno, J. B. Sauvan, Y. Sirois, L. Urda Gómez, E. Vernazza, A. Zabi, A. Zghiche, J.-L. Agram, J. Andrea, D. Apparu, D. Bloch, J.-M. Brom, E. C. Chabert, C. Collard, S. Falke, U. Goerlach, R. Haeberle, A.-C. Le Bihan, M. Meena, O. Poncet, G. Saha, M. A. Sessini, P. Van Hove, P. Vaucelle, A. Di Florio, D. Amram, S. Beauceron, B. Blancon, G. Boudoul, N. Chanon, D. Contardo, P. Depasse, C. Dozen, H. El Mamouni, J. Fay, S. Gascon, M. Gouzevitch, C. Greenberg, G. Grenier, B. Ille, E. Jourd‘huy, I. B. Laktineh, M. Lethuillier, L. Mirabito, S. Perries, A. Purohit, M. Vander Donckt, P. Verdier, J. Xiao, I. Lomidze, T. Toriashvili, Z. Tsamalaidze, V. Botta, S. Consuegra Rodríguez, L. Feld, K. Klein, M. Lipinski, D. Meuser, A. Pauls, D. Pérez Adán, N. Röwert, M. Teroerde, S. Diekmann, A. Dodonova, N. Eich, D. Eliseev, F. Engelke, J. Erdmann, M. Erdmann, P. Fackeldey, B. Fischer, T. Hebbeker, K. Hoepfner, F. Ivone, A. Jung, M.y. Lee, F. Mausolf, M. Merschmeyer, A. Meyer, S. Mukherjee, D. Noll, F. Nowotny, A. Pozdnyakov, Y. Rath, W. Redjeb, F. Rehm, H. Reithler, V. Sarkisovi, A. Schmidt, A. Sharma, J. L. Spah, A. Stein, F. Torres Da Silva De Araujo, S. Wiedenbeck, S. Zaleski, C. Dziwok, G. Flügge, T. Kress, A. Nowack, O. Pooth, A. Stahl, T. Ziemons, A. Zotz, H. Aarup Petersen, M. Aldaya Martin, J. Alimena, S. Amoroso, Y. An, J. Bach, S. Baxter, M. Bayatmakou, H. Becerril Gonzalez, O. Behnke, A. Belvedere, F. Blekman, K. Borras, A. Campbell, A. Cardini, C. Cheng, F. Colombina, M. De Silva, G. Eckerlin, D. Eckstein, L. I. Estevez Banos, O. Filatov, E. Gallo, A. Geiser, V. Guglielmi, M. Guthoff, A. Hinzmann, L. Jeppe, B. Kaech, M. Kasemann, C. Kleinwort, R. Kogler, M. Komm, D. Krücker, W. Lange, D. Leyva Pernia, K. Lipka, W. Lohmann, F. Lorkowski, R. Mankel, I.-A. Melzer-Pellmann, M. Mendizabal Morentin, A. B. Meyer, G. Milella, K. Moral Figueroa, A. Mussgiller, L. P. Nair, J. Niedziela, A. Nürnberg, Y. Otarid, J. Park, E. Ranken, A. Raspereza, D. Rastorguev, J. Rübenach, L. Rygaard, A. Saggio, M. Scham, S. Schnake, P. Schütze, C. Schwanenberger, D. Selivanova, K. Sharko, M. Shchedrolosiev, D. Stafford, F. Vazzoler, A. Ventura Barroso, R. Walsh, D. Wang, Q. Wang, Y. Wen, K. Wichmann, L. Wiens, C. Wissing, Y. Yang, A. Zimermmane Castro Santos, A. Albrecht, S. Albrecht, M. Antonello, S. Bein, L. Benato, S. Bollweg, M. Bonanomi, P. Connor, K. El Morabit, Y. Fischer, E. Garutti, A. Grohsjean, J. Haller, H. R. Jabusch, G. Kasieczka, P. Keicher, R. Klanner, W. Korcari, T. Kramer, C. c. Kuo, V. Kutzner, F. Labe, J. Lange, A. Lobanov, C. Matthies, L. Moureaux, M. Mrowietz, A. Nigamova, Y. Nissan, A. Paasch, K. J. Pena Rodriguez, T. Quadfasel, B. Raciti, M. Rieger, D. Savoiu, J. Schindler, P. Schleper, M. Schröder, J. Schwandt, M. Sommerhalder, H. Stadie, G. Steinbrück, A. Tews, M. Wolf, S. Brommer, M. Burkart, E. Butz, T. Chwalek, A. Dierlamm, A. Droll, U. Elicabuk, N. Faltermann, M. Giffels, A. Gottmann, F. Hartmann, R. Hofsaess, M. Horzela, U. Husemann, J. Kieseler, M. Klute, R. Koppenhöfer, J. M. Lawhorn, M. Link, A. Lintuluoto, B. Maier, S. Maier, S. Mitra, M. Mormile, Th. Müller, M. Neukum, M. Oh, E. Pfeffer, M. Presilla, G. Quast, K. Rabbertz, B. Regnery, N. Shadskiy, I. Shvetsov, H. J. Simonis, L. Sowa, L. Stockmeier, K. Tauqeer, M. Toms, N. Trevisani, R. F. Von Cube, M. Wassmer, S. Wieland, F. Wittig, R. Wolf, X. Zuo, G. Anagnostou, G. Daskalakis, A. Kyriakis, A. Papadopoulos, A. Stakia, P. Kontaxakis, G. Melachroinos, Z. Painesis, I. Papavergou, I. Paraskevas, N. Saoulidou, K. Theofilatos, E. Tziaferi, K. Vellidis, I. Zisopoulos, G. Bakas, T. Chatzistavrou, G. Karapostoli, K. Kousouris, I. Papakrivopoulos, E. Siamarkou, G. Tsipolitis, A. Zacharopoulou, K. Adamidis, I. Bestintzanos, I. Evangelou, C. Foudas, C. Kamtsikis, P. Katsoulis, P. Kokkas, P. G. Kosmoglou Kioseoglou, N. Manthos, I. Papadopoulos, J. Strologas, C. Hajdu, D. Horvath, K. Márton, A. J. Rádl, F. Sikler, V. Veszpremi, M. Csanád, K. Farkas, A. Fehérkuti, M. M. A. Gadallah, Á. Kadlecsik, P. Major, G. Pásztor, G. I. Veres, B. Ujvari, G. Zilizi, G. Bencze, S. Czellar, J. Molnar, Z. Szillasi, F. Nemes, T. Novak, J. Babbar, S. Bansal, S. B. Beri, V. Bhatnagar, G. Chaudhary, S. Chauhan, N. Dhingra, A. Kaur, A. Kaur, H. Kaur, M. Kaur, S. Kumar, K. Sandeep, T. Sheokand, J. B. Singh, A. Singla, A. Ahmed, A. Bhardwaj, A. Chhetri, B. C. Choudhary, A. Kumar, A. Kumar, M. Naimuddin, K. Ranjan, M. K. Saini, S. Saumya, S. Baradia, S. Barman, S. Bhattacharya, S. Das Gupta, S. Dutta, S. Dutta, S. Sarkar, M. M. Ameen, P. K. Behera, S. C. Behera, S. Chatterjee, G. Dash, P. Jana, P. Kalbhor, S. Kamble, J. R. Komaragiri, D. Kumar, P. R. Pujahari, N. R. Saha, A. Sharma, A. K. Sikdar, R. K. Singh, P. Verma, S. Verma, A. Vijay, S. Dugad, G. B. Mohanty, B. Parida, M. Shelake, P. Suryadevara, A. Bala, S. Banerjee, R. M. Chatterjee, M. Guchait, Sh. Jain, A. Jaiswal, S. Kumar, G. Majumder, K. Mazumdar, S. Parolia, A. Thachayath, S. Bahinipati, C. Kar, D. Maity, P. Mal, T. Mishra, V. K. Muraleedharan Nair Bindhu, K. Naskar, A. Nayak, S. Nayak, K. Pal, P. Sadangi, S. K. Swain, S. Varghese, D. Vats, S. Acharya, A. Alpana, S. Dube, B. Gomber, P. Hazarika, B. Kansal, A. Laha, B. Sahu, S. Sharma, K. Y. Vaish, H. Bakhshiansohi, A. Jafari, M. Zeinali, S. Bashiri, S. Chenarani, S. M. Etesami, Y. Hosseini, M. Khakzad, E. Khazaie, M. Mohammadi Najafabadi, S. Tizchang, M. Felcini, M. Grunewald, M. Abbrescia, A. Colaleo, D. Creanza, B. D’Anzi, N. De Filippis, M. De Palma, W. Elmetenawee, L. Fiore, G. Iaselli, L. Longo, M. Louka, G. Maggi, M. Maggi, I. Margjeka, V. Mastrapasqua, S. My, S. Nuzzo, A. Pellecchia, A. Pompili, G. Pugliese, R. Radogna, D. Ramos, A. Ranieri, L. Silvestris, F. M. Simone, Ü. Sözbilir, A. Stamerra, D. Troiano, R. Venditti, P. Verwilligen, A. Zaza, G. Abbiendi, C. Battilana, D. Bonacorsi, P. Capiluppi, A. Castro, F. R. Cavallo, M. Cuffiani, G. M. Dallavalle, T. Diotalevi, F. Fabbri, A. Fanfani, D. Fasanella, P. Giacomelli, L. Giommi, C. Grandi, L. Guiducci, S. Lo Meo, M. Lorusso, L. Lunerti, S. Marcellini, G. Masetti, F. L. Navarria, G. Paggi, A. Perrotta, F. Primavera, A. M. Rossi, S. Rossi Tisbeni, T. Rovelli, G. P. Siroli, S. Costa, A. Di Mattia, A. Lapertosa, R. Potenza, A. Tricomi, C. Tuve, P. Assiouras, G. Barbagli, G. Bardelli, B. Camaiani, A. Cassese, R. Ceccarelli, V. Ciulli, C. Civinini, R. D’Alessandro, E. Focardi, T. Kello, G. Latino, P. Lenzi, M. Lizzo, M. Meschini, S. Paoletti, A. Papanastassiou, G. Sguazzoni, L. Viliani, L. Benussi, S. Bianco, S. Meola, D. Piccolo, P. Chatagnon, F. Ferro, E. Robutti, S. Tosi, A. Benaglia, F. Brivio, F. Cetorelli, F. De Guio, M. E. Dinardo, P. Dini, S. Gennai, R. Gerosa, A. Ghezzi, P. Govoni, L. Guzzi, M. T. Lucchini, M. Malberti, S. Malvezzi, A. Massironi, D. Menasce, L. Moroni, M. Paganoni, S. Palluotto, D. Pedrini, A. Perego, B. S. Pinolini, G. Pizzati, S. Ragazzi, T. Tabarelli de Fatis, S. Buontempo, A. Cagnotta, F. Carnevali, N. Cavallo, F. Fabozzi, A. O. M. Iorio, L. Lista, P. Paolucci, R. Ardino, P. Azzi, N. Bacchetta, M. Bellato, P. Bortignon, G. Bortolato, A. Bragagnolo, A. C. M. Bulla, R. Carlin, P. Checchia, T. Dorigo, U. Gasparini, E. Lusiani, M. Margoni, A. T. Meneguzzo, M. Migliorini, J. Pazzini, P. Ronchese, R. Rossin, M. Sgaravatto, F. Simonetto, M. Tosi, A. Triossi, S. Ventura, M. Zanetti, P. Zotto, A. Zucchetta, G. Zumerle, C. Aimè, A. Braghieri, S. Calzaferri, D. Fiorina, P. Montagna, V. Re, C. Riccardi, P. Salvini, I. Vai, P. Vitulo, S. Ajmal, M. E. Ascioti, G. M. Bilei, C. Carrivale, D. Ciangottini, L. Fanò, M. Magherini, V. Mariani, M. Menichelli, F. Moscatelli, A. Rossi, A. Santocchia, D. Spiga, T. Tedeschi, C. A. Alexe, P. Asenov, P. Azzurri, G. Bagliesi, R. Bhattacharya, L. Bianchini, T. Boccali, E. Bossini, D. Bruschini, R. Castaldi, M. A. Ciocci, M. Cipriani, V. D’Amante, R. Dell’Orso, S. Donato, A. Giassi, F. Ligabue, A. C. Marini, D. Matos Figueiredo, A. Messineo, M. Musich, F. Palla, A. Rizzi, G. Rolandi, S. Roy Chowdhury, T. Sarkar, A. Scribano, P. Spagnolo, R. Tenchini, G. Tonelli, N. Turini, F. Vaselli, A. Venturi, P. G. Verdini, C. Baldenegro Barrera, P. Barria, C. Basile, M. Campana, F. Cavallari, L. Cunqueiro Mendez, D. Del Re, E. Di Marco, M. Diemoz, F. Errico, E. Longo, J. Mijuskovic, G. Organtini, F. Pandolfi, R. Paramatti, C. Quaranta, S. Rahatlou, C. Rovelli, F. Santanastasio, L. Soffi, N. Amapane, R. Arcidiacono, S. Argiro, M. Arneodo, N. Bartosik, R. Bellan, A. Bellora, C. Biino, C. Borca, N. Cartiglia, M. Costa, R. Covarelli, N. Demaria, L. Finco, M. Grippo, B. Kiani, F. Legger, F. Luongo, C. Mariotti, L. Markovic, S. Maselli, A. Mecca, L. Menzio, P. Meridiani, E. Migliore, M. Monteno, R. Mulargia, M. M. Obertino, G. Ortona, L. Pacher, N. Pastrone, M. Pelliccioni, M. Ruspa, F. Siviero, V. Sola, A. Solano, A. Staiano, C. Tarricone, D. Trocino, G. Umoret, R. White, S. Belforte, V. Candelise, M. Casarsa, F. Cossutti, K. De Leo, G. Della Ricca, S. Dogra, J. Hong, C. Huh, B. Kim, J. Kim, D. Lee, H. Lee, S. W. Lee, C. S. Moon, Y. D. Oh, M. S. Ryu, S. Sekmen, B. Tae, Y. C. Yang, M. S. Kim, G. Bak, P. Gwak, H. Kim, D. H. Moon, E. Asilar, J. Choi, D. Kim, T. J. Kim, J. A. Merlin, Y. Ryou, S. Choi, S. Han, B. Hong, K. Lee, K. S. Lee, S. Lee, J. Yoo, J. Goh, S. Yang, H. S. Kim, Y. Kim, S. Lee, J. Almond, J. H. Bhyun, J. Choi, J. Choi, W. Jun, J. Kim, S. Ko, H. Kwon, H. Lee, J. Lee, J. Lee, B. H. Oh, S. B. Oh, H. Seo, U. K. Yang, I. Yoon, W. Jang, D. Y. Kang, Y. Kang, S. Kim, B. Ko, J. S. H. Lee, Y. Lee, I. C. Park, Y. Roh, I. J. Watson, S. Ha, H. D. Yoo, M. Choi, M. R. Kim, H. Lee, Y. Lee, I. Yu, T. Beyrouthy, Y. Gharbia, K. Dreimanis, A. Gaile, C. Munoz Diaz, D. Osite, G. Pikurs, A. Potrebko, M. Seidel, D. Sidiropoulos Kontos, N. R. Strautnieks, M. Ambrozas, A. Juodagalvis, A. Rinkevicius, G. Tamulaitis, I. Yusuff, Z. Zolkapli, J. F. Benitez, A. Castaneda Hernandez, H. A. Encinas Acosta, L. G. Gallegos Maríñez, M. León Coello, J. A. Murillo Quijada, A. Sehrawat, L. Valencia Palomo, G. Ayala, H. Castilla-Valdez, H. Crotte Ledesma, E. De La Cruz-Burelo, I. Heredia-De La Cruz, R. Lopez-Fernandez, J. Mejia Guisao, C. A. Mondragon Herrera, A. Sánchez Hernández, C. Oropeza Barrera, D. L. Ramirez Guadarrama, M. Ramírez García, I. Bautista, I. Pedraza, H. A. Salazar Ibarguen, C. Uribe Estrada, I. Bubanja, N. Raicevic, P. H. Butler, A. Ahmad, M. I. Asghar, A. Awais, M. I. M. Awan, H. R. Hoorani, W. A. Khan, V. Avati, L. Grzanka, M. Malawski, H. Bialkowska, M. Bluj, M. Górski, M. Kazana, M. Szleper, P. Zalewski, K. Bunkowski, K. Doroba, A. Kalinowski, M. Konecki, J. Krolikowski, A. Muhammad, K. Pozniak, W. Zabolotny, M. Araujo, D. Bastos, C. Beirão Da Cruz E Silva, A. Boletti, M. Bozzo, T. Camporesi, G. Da Molin, M. Gallinaro, J. Hollar, N. Leonardo, G. B. Marozzo, T. Niknejad, A. Petrilli, M. Pisano, J. Seixas, J. Varela, J. W. Wulff, P. Adzic, P. Milenovic, M. Dordevic, J. Milosevic, V. Rekovic, J. Alcaraz Maestre, Cristina F. Bedoya, Oliver M. Carretero, M. Cepeda, M. Cerrada, N. Colino, B. De La Cruz, A. Delgado Peris, A. Escalante Del Valle, D. Fernández Del Val, J. P. Fernández Ramos, J. Flix, M. C. Fouz, O. Gonzalez Lopez, S. Goy Lopez, J. M. Hernandez, M. I. Josa, E. Martin Viscasillas, D. Moran, C. M. Morcillo Perez, Á. Navarro Tobar, C. Perez Dengra, A. Pérez-Calero Yzquierdo, J. Puerta Pelayo, I. Redondo, S. Sánchez Navas, J. Sastre, J. Vazquez Escobar, J. F. de Trocóniz, B. Alvarez Gonzalez, J. Cuevas, J. Fernandez Menendez, S. Folgueras, I. Gonzalez Caballero, J. R. González Fernández, P. Leguina, E. Palencia Cortezon, J. Prado Pico, C. Ramón Álvarez, V. Rodríguez Bouza, A. Soto Rodríguez, A. Trapote, C. Vico Villalba, P. Vischia, S. Bhowmik, S. Blanco Fernández, J. A. Brochero Cifuentes, I. J. Cabrillo, A. Calderon, J. Duarte Campderros, M. Fernandez, G. Gomez, C. Lasaosa García, R. Lopez Ruiz, C. Martinez Rivero, P. Martinez Ruiz del Arbol, F. Matorras, P. Matorras Cuevas, E. Navarrete Ramos, J. Piedra Gomez, L. Scodellaro, I. Vila, J. M. Vizan Garcia, B. Kailasapathy, D. D. C. Wickramarathna, W. G. D. Dharmaratna, K. Liyanage, N. Perera, D. Abbaneo, C. Amendola, E. Auffray, G. Auzinger, J. Baechler, D. Barney, A. Bermúdez Martínez, M. Bianco, A. A. Bin Anuar, A. Bocci, L. Borgonovi, C. Botta, E. Brondolin, C. Caillol, G. Cerminara, N. Chernyavskaya, D. d’Enterria, A. Dabrowski, A. David, A. De Roeck, M. M. Defranchis, M. Deile, M. Dobson, G. Franzoni, W. Funk, S. Giani, D. Gigi, K. Gill, F. Glege, J. Hegeman, J. K. Heikkilä, B. Huber, V. Innocente, T. James, P. Janot, O. Kaluzinska, O. Karacheban, S. Laurila, P. Lecoq, E. Leutgeb, C. Lourenço, L. Malgeri, M. Mannelli, M. Matthewman, A. Mehta, F. Meijers, S. Mersi, E. Meschi, V. Milosevic, F. Monti, F. Moortgat, M. Mulders, I. Neutelings, S. Orfanelli, F. Pantaleo, G. Petrucciani, A. Pfeiffer, M. Pierini, H. Qu, D. Rabady, B. Ribeiro Lopes, M. Rovere, H. Sakulin, S. Sanchez Cruz, S. Scarfi, C. Schwick, M. Selvaggi, A. Sharma, K. Shchelina, P. Silva, P. Sphicas, A. G. Stahl Leiton, A. Steen, S. Summers, D. Treille, P. Tropea, D. Walter, J. Wanczyk, J. Wang, S. Wuchterl, P. Zehetner, P. Zejdl, W. D. Zeuner, T. Bevilacqua, L. Caminada, A. Ebrahimi, W. Erdmann, R. Horisberger, Q. Ingram, H. C. Kaestli, D. Kotlinski, C. Lange, M. Missiroli, L. Noehte, T. Rohe, T. K. Aarrestad, K. Androsov, M. Backhaus, G. Bonomelli, A. Calandri, C. Cazzaniga, K. Datta, P. De Bryas Dexmiers D‘archiac, A. De Cosa, G. Dissertori, M. Dittmar, M. Donegà, F. Eble, M. Galli, K. Gedia, F. Glessgen, C. Grab, N. Härringer, T. G. Harte, D. Hits, W. Lustermann, A.-M. Lyon, R. A. Manzoni, M. Marchegiani, L. Marchese, C. Martin Perez, A. Mascellani, F. Nessi-Tedaldi, F. Pauss, V. Perovic, S. Pigazzini, C. Reissel, B. Ristic, F. Riti, R. Seidita, J. Steggemann, A. Tarabini, D. Valsecchi, R. Wallny, C. Amsler, P. Bärtschi, M. F. Canelli, K. Cormier, M. Huwiler, W. Jin, A. Jofrehei, B. Kilminster, S. Leontsinis, S. P. Liechti, A. Macchiolo, P. Meiring, F. Meng, U. Molinatti, J. Motta, A. Reimers, P. Robmann, M. Senger, E. Shokr, F. Stäger, R. Tramontano, C. Adloff, D. Bhowmik, C. M. Kuo, W. Lin, P. K. Rout, P. C. Tiwari, S. S. Yu, L. Ceard, K. F. Chen, P. s. Chen, Z. g. Chen, A. De Iorio, W.-S. Hou, T. h. Hsu, Y. w. Kao, S. Karmakar, G. Kole, Y.y. Li, R.-S. Lu, E. Paganis, X.f. Su, J. Thomas-Wilsker, L. s. Tsai, D. Tsionou, H. y. Wu, E. Yazgan, C. Asawatangtrakuldee, N. Srimanobhas, V. Wachirapusitanand, D. Agyel, F. Boran, F. Dolek, I. Dumanoglu, E. Eskut, Y. Guler, E. Gurpinar Guler, C. Isik, O. Kara, A. Kayis Topaksu, U. Kiminsu, G. Onengut, K. Ozdemir, A. Polatoz, B. Tali, U. G. Tok, S. Turkcapar, E. Uslan, I. S. Zorbakir, G. Sokmen, M. Yalvac, B. Akgun, I. O. Atakisi, E. Gülmez, M. Kaya, O. Kaya, S. Tekten, A. Cakir, K. Cankocak, G. G. Dincer, Y. Komurcu, S. Sen, O. Aydilek, B. Hacisahinoglu, I. Hos, B. Kaynak, S. Ozkorucuklu, O. Potok, H. Sert, C. Simsek, C. Zorbilmez, S. Cerci, B. Isildak, D. Sunar Cerci, T. Yetkin, A. Boyaryntsev, B. Grynyov, L. Levchuk, D. Anthony, J. J. Brooke, A. Bundock, F. Bury, E. Clement, D. Cussans, H. Flacher, M. Glowacki, J. Goldstein, H. F. Heath, M.-L. Holmberg, L. Kreczko, S. Paramesvaran, L. Robertshaw, S. Seif El Nasr-Storey, V. J. Smith, N. Stylianou, K. Walkingshaw Pass, A. H. Ball, K. W. Bell, A. Belyaev, C. Brew, R. M. Brown, D. J. A. Cockerill, C. Cooke, A. Elliot, K. V. Ellis, K. Harder, S. Harper, J. Linacre, K. Manolopoulos, D. M. Newbold, E. Olaiya, D. Petyt, T. Reis, A. R. Sahasransu, G. Salvi, T. Schuh, C. H. Shepherd-Themistocleous, I. R. Tomalin, K. C. Whalen, T. Williams, I. Andreou, R. Bainbridge, P. Bloch, C. E. Brown, O. Buchmuller, V. Cacchio, C. A. Carrillo Montoya, G. S. Chahal, D. Colling, J. S. Dancu, I. Das, P. Dauncey, G. Davies, J. Davies, M. Della Negra, S. Fayer, G. Fedi, G. Hall, M. H. Hassanshahi, A. Howard, G. Iles, C. R. Knight, J. Langford, J. León Holgado, L. Lyons, A.-M. Magnan, S. Mallios, M. Mieskolainen, J. Nash, M. Pesaresi, P. B. Pradeep, B. C. Radburn-Smith, A. Richards, A. Rose, K. Savva, C. Seez, R. Shukla, A. Tapper, K. Uchida, G. P. Uttley, L. H. Vage, T. Virdee, M. Vojinovic, N. Wardle, D. Winterbottom, K. Coldham, J. E. Cole, A. Khan, P. Kyberd, I. D. Reid, S. Abdullin, A. Brinkerhoff, E. Collins, J. Dittmann, K. Hatakeyama, J. Hiltbrand, B. McMaster, J. Samudio, S. Sawant, C. Sutantawibul, J. Wilson, R. Bartek, A. Dominguez, C. Huerta Escamilla, A. E. Simsek, R. Uniyal, A. M. Vargas Hernandez, B. Bam, A. Buchot Perraguin, R. Chudasama, S. I. Cooper, C. Crovella, S. V. Gleyzer, E. Pearson, C. U. Perez, P. Rumerio, E. Usai, R. Yi, A. Akpinar, C. Cosby, G. De Castro, Z. Demiragli, C. Erice, C. Fangmeier, C. Fernandez Madrazo, E. Fontanesi, D. Gastler, F. Golf, S. Jeon, J. O‘cain, I. Reed, J. Rohlf, K. Salyer, D. Sperka, D. Spitzbart, I. Suarez, A. Tsatsos, A. G. Zecchinelli, G. Benelli, D. Cutts, L. Gouskos, M. Hadley, U. Heintz, J. M. Hogan, T. Kwon, G. Landsberg, K. T. Lau, D. Li, J. Luo, S. Mondal, N. Pervan, T. Russell, S. Sagir, F. Simpson, M. Stamenkovic, N. Venkatasubramanian, X. Yan, S. Abbott, C. Brainerd, R. Breedon, H. Cai, M. Calderon De La Barca Sanchez, M. Chertok, M. Citron, J. Conway, P. T. Cox, R. Erbacher, F. Jensen, O. Kukral, G. Mocellin, M. Mulhearn, S. Ostrom, W. Wei, Y. Yao, S. Yoo, F. Zhang, M. Bachtis, R. Cousins, A. Datta, G. Flores Avila, J. Hauser, M. Ignatenko, M. A. Iqbal, T. Lam, E. Manca, A. Nunez Del Prado, D. Saltzberg, V. Valuev, R. Clare, J. W. Gary, M. Gordon, G. Hanson, W. Si, A. Aportela, A. Arora, J. G. Branson, S. Cittolin, S. Cooperstein, D. Diaz, J. Duarte, L. Giannini, Y. Gu, J. Guiang, R. Kansal, V. Krutelyov, R. Lee, J. Letts, M. Masciovecchio, F. Mokhtar, S. Mukherjee, M. Pieri, M. Quinnan, B. V. Sathia Narayanan, V. Sharma, M. Tadel, E. Vourliotis, F. Würthwein, Y. Xiang, A. Yagil, A. Barzdukas, L. Brennan, C. Campagnari, K. Downham, C. Grieco, J. Incandela, J. Kim, A. J. Li, P. Masterson, H. Mei, J. Richman, S. N. Santpur, U. Sarica, R. Schmitz, F. Setti, J. Sheplock, D. Stuart, T. Á. Vámi, S. Wang, D. Zhang, S. Bhattacharya, A. Bornheim, O. Cerri, A. Latorre, J. Mao, H. B. Newman, G. Reales Gutiérrez, M. Spiropulu, J. R. Vlimant, C. Wang, S. Xie, R. Y. Zhu, J. Alison, S. An, P. Bryant, M. Cremonesi, V. Dutta, T. Ferguson, T. A. Gómez Espinosa, A. Harilal, A. Kallil Tharayil, C. Liu, T. Mudholkar, S. Murthy, P. Palit, K. Park, M. Paulini, A. Roberts, A. Sanchez, W. Terrill, J. P. Cumalat, W. T. Ford, A. Hart, A. Hassani, G. Karathanasis, N. Manganelli, J. Pearkes, C. Savard, N. Schonbeck, K. Stenson, K. A. Ulmer, S. R. Wagner, N. Zipper, D. Zuolo, J. Alexander, S. Bright-Thonney, X. Chen, D. J. Cranshaw, J. Fan, X. Fan, S. Hogan, P. Kotamnives, J. Monroy, M. Oshiro, J. R. Patterson, M. Reid, A. Ryd, J. Thom, P. Wittich, R. Zou, M. Albrow, M. Alyari, O. Amram, G. Apollinari, A. Apresyan, L. A. T. Bauerdick, D. Berry, J. Berryhill, P. C. Bhat, K. Burkett, J. N. Butler, A. Canepa, G. B. Cerati, H. W. K. Cheung, F. Chlebana, G. Cummings, J. Dickinson, I. Dutta, V. D. Elvira, Y. Feng, J. Freeman, A. Gandrakota, Z. Gecse, L. Gray, D. Green, A. Grummer, S. Grünendahl, D. Guerrero, O. Gutsche, R. M. Harris, R. Heller, T. C. Herwig, J. Hirschauer, B. Jayatilaka, S. Jindariani, M. Johnson, U. Joshi, T. Klijnsma, B. Klima, K. H. M. Kwok, S. Lammel, D. Lincoln, R. Lipton, T. Liu, C. Madrid, K. Maeshima, C. Mantilla, D. Mason, P. McBride, P. Merkel, S. Mrenna, S. Nahn, J. Ngadiuba, D. Noonan, S. Norberg, V. Papadimitriou, N. Pastika, K. Pedro, C. Pena, F. Ravera, A. Reinsvold Hall, L. Ristori, M. Safdari, E. Sexton-Kennedy, N. Smith, A. Soha, L. Spiegel, S. Stoynev, J. Strait, L. Taylor, S. Tkaczyk, N. V. Tran, L. Uplegger, E. W. Vaandering, I. Zoi, C. Aruta, P. Avery, D. Bourilkov, P. Chang, V. Cherepanov, R. D. Field, E. Koenig, M. Kolosova, J. Konigsberg, A. Korytov, K. Matchev, N. Menendez, G. Mitselmakher, K. Mohrman, A. Muthirakalayil Madhu, N. Rawal, S. Rosenzweig, Y. Takahashi, J. Wang, T. Adams, A. Al Kadhim, A. Askew, S. Bower, V. Hagopian, R. Hashmi, R. S. Kim, S. Kim, T. Kolberg, G. Martinez, H. Prosper, P. R. Prova, M. Wulansatiti, R. Yohay, J. Zhang, B. Alsufyani, M. M. Baarmand, S. Butalla, S. Das, T. Elkafrawy, M. Hohlmann, E. Yanes, M. R. Adams, A. Baty, C. Bennett, R. Cavanaugh, R. Escobar Franco, O. Evdokimov, C. E. Gerber, M. Hawksworth, A. Hingrajiya, D. J. Hofman, J.h. Lee, D. S. Lemos, A. H. Merrit, C. Mills, S. Nanda, G. Oh, B. Ozek, D. Pilipovic, R. Pradhan, E. Prifti, T. Roy, S. Rudrabhatla, M. B. Tonjes, N. Varelas, M. A. Wadud, Z. Ye, J. Yoo, M. Alhusseini, D. Blend, K. Dilsiz, L. Emediato, G. Karaman, O. K. Köseyan, J.-P. Merlo, A. Mestvirishvili, O. Neogi, H. Ogul, Y. Onel, A. Penzo, C. Snyder, E. Tiras, B. Blumenfeld, L. Corcodilos, J. Davis, A. V. Gritsan, L. Kang, S. Kyriacou, P. Maksimovic, M. Roguljic, J. Roskes, S. Sekhar, M. Swartz, A. Abreu, L. F. Alcerro Alcerro, J. Anguiano, S. Arteaga Escatel, P. Baringer, A. Bean, Z. Flowers, D. Grove, J. King, G. Krintiras, M. Lazarovits, C. Le Mahieu, J. Marquez, M. Murray, M. Nickel, M. Pitt, S. Popescu, C. Rogan, C. Royon, R. Salvatico, S. Sanders, C. Smith, G. Wilson, B. Allmond, R. Gujju Gurunadha, A. Ivanov, K. Kaadze, Y. Maravin, J. Natoli, D. Roy, G. Sorrentino, A. Baden, A. Belloni, J. Bistany-riebman, Y. M. Chen, S. C. Eno, N. J. Hadley, S. Jabeen, R. G. Kellogg, T. Koeth, B. Kronheim, Y. Lai, S. Lascio, A. C. Mignerey, S. Nabili, C. Palmer, C. Papageorgakis, M. M. Paranjpe, L. Wang, J. Bendavid, I. A. Cali, P.c. Chou, M. D’Alfonso, J. Eysermans, C. Freer, G. Gomez-Ceballos, M. Goncharov, G. Grosso, P. Harris, D. Hoang, D. Kovalskyi, J. Krupa, L. Lavezzo, Y.-J. Lee, K. Long, C. Mcginn, A. Novak, C. Paus, C. Roland, G. Roland, S. Rothman, G. S. F. Stephans, Z. Wang, B. Wyslouch, T. J. Yang, B. Crossman, B. M. Joshi, C. Kapsiak, M. Krohn, D. Mahon, J. Mans, B. Marzocchi, R. Rusack, R. Saradhy, N. Strobbe, K. Bloom, D. R. Claes, G. Haza, J. Hossain, C. Joo, I. Kravchenko, J. E. Siado, W. Tabb, A. Vagnerini, A. Wightman, F. Yan, D. Yu, H. Bandyopadhyay, L. Hay, H. w. Hsia, I. Iashvili, A. Kalogeropoulos, A. Kharchilava, M. Morris, D. Nguyen, S. Rappoccio, H. Rejeb Sfar, A. Williams, P. Young, G. Alverson, E. Barberis, J. Bonilla, J. Dervan, Y. Haddad, Y. Han, A. Krishna, J. Li, M. Lu, G. Madigan, R. Mccarthy, D. M. Morse, V. Nguyen, T. Orimoto, A. Parker, L. Skinnari, D. Wood, J. Bueghly, S. Dittmer, K. A. Hahn, Y. Liu, Y. Miao, D. G. Monk, M. H. Schmitt, A. Taliercio, M. Velasco, G. Agarwal, R. Band, R. Bucci, S. Castells, A. Das, R. Goldouzian, M. Hildreth, K. W. Ho, K. Hurtado Anampa, T. Ivanov, C. Jessop, K. Lannon, J. Lawrence, N. Loukas, L. Lutton, J. Mariano, N. Marinelli, I. Mcalister, T. McCauley, C. Mcgrady, C. Moore, Y. Musienko, H. Nelson, M. Osherson, A. Piccinelli, R. Ruchti, A. Townsend, Y. Wan, M. Wayne, H. Yockey, M. Zarucki, L. Zygala, A. Basnet, B. Bylsma, M. Carrigan, L. S. Durkin, C. Hill, M. Joyce, M. Nunez Ornelas, K. Wei, B. L. Winer, B. R. Yates, H. Bouchamaoui, P. Das, G. Dezoort, P. Elmer, A. Frankenthal, B. Greenberg, N. Haubrich, K. Kennedy, G. Kopp, S. Kwan, D. Lange, A. Loeliger, D. Marlow, I. Ojalvo, J. Olsen, A. Shevelev, D. Stickland, C. Tully, S. Malik, A. S. Bakshi, S. Chandra, R. Chawla, A. Gu, L. Gutay, M. Jones, A. W. Jung, A. M. Koshy, M. Liu, G. Negro, N. Neumeister, G. Paspalaki, S. Piperov, V. Scheurer, J. F. Schulte, M. Stojanovic, J. Thieman, A. K. Virdi, F. Wang, W. Xie, J. Dolen, N. Parashar, A. Pathak, D. Acosta, T. Carnahan, K. M. Ecklund, P. J. Fernández Manteca, S. Freed, P. Gardner, F. J. M. Geurts, I. Krommydas, W. Li, J. Lin, O. Miguel Colin, B. P. Padley, R. Redjimi, J. Rotter, E. Yigitbasi, Y. Zhang, A. Bodek, P. de Barbaro, R. Demina, A. Garcia-Bellido, O. Hindrichs, A. Khukhunaishvili, N. Parmar, P. Parygin, E. Popova, R. Taus, B. Chiarito, J. P. Chou, S. V. Clark, D. Gadkari, Y. Gershtein, E. Halkiadakis, M. Heindl, C. Houghton, D. Jaroslawski, S. Konstantinou, I. Laflotte, A. Lath, R. Montalvo, K. Nash, J. Reichert, H. Routray, P. Saha, S. Salur, S. Schnetzer, S. Somalwar, R. Stone, S. A. Thayil, S. Thomas, J. Vora, H. Wang, D. Ally, A. G. Delannoy, S. Fiorendi, S. Higginbotham, T. Holmes, A. R. Kanuganti, N. Karunarathna, L. Lee, E. Nibigira, S. Spanier, D. Aebi, M. Ahmad, T. Akhter, O. Bouhali, R. Eusebi, J. Gilmore, T. Huang, T. Kamon, H. Kim, S. Luo, R. Mueller, D. Overton, D. Rathjens, A. Safonov, N. Akchurin, J. Damgov, N. Gogate, V. Hegde, A. Hussain, Y. Kazhykarim, K. Lamichhane, S. W. Lee, A. Mankel, T. Peltola, I. Volobouev, E. Appelt, Y. Chen, S. Greene, A. Gurrola, W. Johns, R. Kunnawalkam Elayavalli, A. Melo, F. Romeo, P. Sheldon, S. Tuo, J. Velkovska, J. Viinikainen, B. Cardwell, H. Chung, B. Cox, J. Hakala, R. Hirosky, A. Ledovskoy, C. Neu, S. Bhattacharya, P. E. Karchin, A. Aravind, S. Banerjee, K. Black, T. Bose, S. Dasu, I. De Bruyn, P. Everaerts, C. Galloni, H. He, M. Herndon, A. Herve, C. K. Koraka, A. Lanaro, R. Loveless, J. Madhusudanan Sreekala, A. Mallampalli, A. Mohammadi, S. Mondal, G. Parida, L. Pétré, D. Pinna, A. Savin, V. Shang, V. Sharma, W. H. Smith, D. Teague, H. F. Tsoi, W. Vetens, A. Warden, S. Afanasiev, V. Alexakhin, V. Andreev, Yu. Andreev, T. Aushev, M. Azarkin, A. Babaev, V. Blinov, E. Boos, V. Borshch, D. Budkouski, V. Bunichev, V. Chekhovsky, R. Chistov, M. Danilov, A. Dermenev, T. Dimova, D. Druzhkin, M. Dubinin, L. Dudko, A. Ershov, G. Gavrilov, V. Gavrilov, S. Gninenko, V. Golovtcov, N. Golubev, I. Golutvin, I. Gorbunov, A. Gribushin, Y. Ivanov, V. Kachanov, V. Karjavine, A. Karneyeu, V. Kim, M. Kirakosyan, D. Kirpichnikov, M. Kirsanov, V. Klyukhin, O. Kodolova, D. Konstantinov, V. Korenkov, A. Kozyrev, N. Krasnikov, A. Lanev, P. Levchenko, N. Lychkovskaya, V. Makarenko, A. Malakhov, V. Matveev, V. Murzin, A. Nikitenko, S. Obraztsov, V. Oreshkin, V. Palichik, V. Perelygin, S. Petrushanko, S. Polikarpov, V. Popov, O. Radchenko, M. Savina, V. Savrin, V. Sergeychik, V. Shalaev, S. Shmatov, S. Shulha, Y. Skovpen, S. Slabospitskii, V. Smirnov, A. Snigirev, D. Sosnov, V. Sulimov, A. Terkulov, O. Teryaev, I. Tlisova, A. Toropin, A. Tulupov, L. Uvarov, A. Uzunian, A. Vorobyev, N. Voytishin, B. S. Yuldashev, A. Zarubin, I. Zhizhin, A. Zhokin

**Affiliations:** 1 Yerevan Physics Institute, Yerevan, Armenia; 2 Institut für Hochenergiephysik, Vienna, Austria; 3 Universiteit Antwerpen, Antwerpen, Belgium; 4 Vrije Universiteit Brussel, Brussel, Belgium; 5 Université Libre de Bruxelles, Bruxelles, Belgium; 6 Ghent University, Ghent, Belgium; 7 Université Catholique de Louvain, Louvain-la-Neuve, Belgium; 8 Centro Brasileiro de Pesquisas Fisicas, Rio de Janeiro, Brazil; 9 Universidade do Estado do Rio de Janeiro, Rio de Janeiro, Brazil; 10 Universidade Estadual Paulista, Universidade Federal do ABC, São Paulo, Brazil; 11 Institute for Nuclear Research and Nuclear Energy, Bulgarian Academy of Sciences, Sofia, Bulgaria; 12 University of Sofia, Sofia, Bulgaria; 13 Instituto De Alta Investigación, Universidad de Tarapacá, Casilla 7 D, Arica, Chile; 14 Beihang University, Beijing, China; 15 Department of Physics, Tsinghua University, Beijing, China; 16 Institute of High Energy Physics, Beijing, China; 17 State Key Laboratory of Nuclear Physics and Technology, Peking University, Beijing, China; 18 Guangdong Provincial Key Laboratory of Nuclear Science and Guangdong-Hong Kong Joint Laboratory of Quantum Matter, South China Normal University, Guangzhou, China; 19 Sun Yat-Sen University, Guangzhou, China; 20 University of Science and Technology of China, Hefei, China; 21 Nanjing Normal University, Nanjing, China; 22 Institute of Modern Physics and Key Laboratory of Nuclear Physics and Ion-beam Application (MOE), Fudan University, Shanghai, China; 23 Zhejiang University, Hangzhou, Zhejiang, China; 24 Universidad de Los Andes, Bogotá, Colombia; 25 Universidad de Antioquia, Medellín, Colombia; 26 Faculty of Electrical Engineering, Mechanical Engineering and Naval Architecture, University of Split, Split, Croatia; 27 University of Split, Faculty of Science, Split, Croatia; 28 Institute Rudjer Boskovic, Zagreb, Croatia; 29 University of Cyprus, Nicosia, Cyprus; 30 Charles University, Prague, Czech Republic; 31 Universidad San Francisco de Quito, Quito, Ecuador; 32 Academy of Scientific Research and Technology of the Arab Republic of Egypt, Egyptian Network of High Energy Physics, Cairo, Egypt; 33 Center for High Energy Physics (CHEP-FU), Fayoum University, El-Fayoum, Egypt; 34 National Institute of Chemical Physics and Biophysics, Tallinn, Estonia; 35 Department of Physics, University of Helsinki, Helsinki, Finland; 36 Helsinki Institute of Physics, Helsinki, Finland; 37 Lappeenranta-Lahti University of Technology, Lappeenranta, Finland; 38 IRFU, CEA, Université Paris-Saclay, Gif-sur-Yvette, France; 39 Laboratoire Leprince-Ringuet, CNRS/IN2P3, Ecole Polytechnique, Institut Polytechnique de Paris, Palaiseau, France; 40 Université de Strasbourg, CNRS, IPHC UMR 7178, Strasbourg, France; 41 Centre de Calcul de l’Institut National de Physique Nucleaire et de Physique des Particules, CNRS/IN2P3, Villeurbanne, France; 42 Institut de Physique des 2 Infinis de Lyon (IP2I ), Villeurbanne, France; 43 Georgian Technical University, Tbilisi, Georgia; 44 I. Physikalisches Institut, RWTH Aachen University, Aachen, Germany; 45 III. Physikalisches Institut A, RWTH Aachen University, Aachen, Germany; 46 III. Physikalisches Institut B, RWTH Aachen University, Aachen, Germany; 47 Deutsches Elektronen-Synchrotron, Hamburg, Germany; 48 University of Hamburg, Hamburg, Germany; 49 Karlsruher Institut fuer Technologie, Karlsruhe, Germany; 50 Institute of Nuclear and Particle Physics (INPP), NCSR Demokritos, Aghia Paraskevi, Greece; 51 National and Kapodistrian University of Athens, Athens, Greece; 52 National Technical University of Athens, Athens, Greece; 53 University of Ioánnina, Ioannina, Greece; 54 HUN-REN Wigner Research Centre for Physics, Budapest, Hungary; 55 MTA-ELTE Lendület CMS Particle and Nuclear Physics Group, Eötvös Loránd University, Budapest, Hungary; 56 Faculty of Informatics, University of Debrecen, Debrecen, Hungary; 57 Institute of Nuclear Research ATOMKI, Debrecen, Hungary; 58 Karoly Robert Campus, MATE Institute of Technology, Gyongyos, Hungary; 59 Panjab University, Chandigarh, India; 60 University of Delhi, Delhi, India; 61 Saha Institute of Nuclear Physics, HBNI, Kolkata, India; 62 Indian Institute of Technology Madras, Madras, India; 63 Tata Institute of Fundamental Research-A, Mumbai, India; 64 Tata Institute of Fundamental Research-B, Mumbai, India; 65 National Institute of Science Education and Research, An OCC of Homi Bhabha National Institute, Bhubaneswar, Odisha India; 66 Indian Institute of Science Education and Research (IISER), Pune, India; 67 Isfahan University of Technology, Isfahan, Iran; 68 Institute for Research in Fundamental Sciences (IPM), Tehran, Iran; 69 University College Dublin, Dublin, Ireland; 70 INFN Sezione di Bari,Università di Bari, Politecnico di Bari, Bari, Italy; 71 INFN Sezione di Bologna, Università di Bologna, Bologna, Italy; 72 INFN Sezione di Catania, Università di Catania, Catania, Italy; 73 INFN Sezione di Firenze,Università di Firenze, Firenze, Italy; 74 INFN Laboratori Nazionali di Frascati, Frascati, Italy; 75 INFN Sezione di Genova,Università di Genova, Genoa, Italy; 76 INFN Sezione di Milano-Bicocca,Università di Milano-Bicocca, Milan, Italy; 77 INFN Sezione di Napoli, Università di Napoli ’Federico II’, Napoli, Italy; Università della Basilicata, Potenza, Italy; Scuola Superiore Meridionale (SSM), Naples, Italy; 78 INFN Sezione di Padova, Università di Padova, Padova, Italy; Università di Trento, Trento, Italy; 79 INFN Sezione di Pavia, Università di Pavia, Pavia, Italy; 80 INFN Sezione di Perugia, Università di Perugia, Perugia, Italy; 81 INFN Sezione di Pisa, Università di Pisa, Scuola Normale Superiore di Pisa, Pisa, Italy; Università di Siena, Siena, Italy; 82 INFN Sezione di Roma, Sapienza Università di Roma, Rome, Italy; 83 INFN Sezione di Torino, Università di Torino, Torino, Italy; Università del Piemonte Orientale, Novara, Italy; 84 INFN Sezione di Trieste, Università di Trieste, Trieste, Italy; 85 Kyungpook National University, Daegu, Korea; 86 Department of Mathematics and Physics-GWNU, Gangneung, Korea; 87 Chonnam National University, Institute for Universe and Elementary Particles, Kwangju, Korea; 88 Hanyang University, Seoul, Korea; 89 Korea University, Seoul, Korea; 90 Department of Physics, Kyung Hee University, Seoul, Korea; 91 Sejong University, Seoul, Korea; 92 Seoul National University, Seoul, Korea; 93 University of Seoul, Seoul, Korea; 94 Department of Physics, Yonsei University, Seoul, Korea; 95 Sungkyunkwan University, Suwon, Korea; 96 College of Engineering and Technology, American University of the Middle East (AUM), Dasman, Kuwait; 97 Riga Technical University, Riga, Latvia; 98 University of Latvia (LU), Riga, Latvia; 99 Vilnius University, Vilnius, Lithuania; 100 National Centre for Particle Physics, Universiti Malaya, Kuala Lumpur, Malaysia; 101 Universidad de Sonora (UNISON), Hermosillo, Mexico; 102 Centro de Investigacion y de Estudios Avanzados del IPN, Mexico City, Mexico; 103 Universidad Iberoamericana, Mexico City, Mexico; 104 Benemerita Universidad Autonoma de Puebla, Puebla, Mexico; 105 University of Montenegro, Podgorica, Montenegro; 106 University of Canterbury, Christchurch, New Zealand; 107 National Centre for Physics, Quaid-I-Azam University, Islamabad, Pakistan; 108 Faculty of Computer Science, Electronics and Telecommunications, AGH University of Krakow, Kraków, Poland; 109 National Centre for Nuclear Research, Swierk, Poland; 110 Institute of Experimental Physics, Faculty of Physics, University of Warsaw, Warsaw, Poland; 111 Warsaw University of Technology, Warsaw, Poland; 112 Laboratório de Instrumentação e Física Experimental de Partículas, Lisbon, Portugal; 113 Faculty of Physics, University of Belgrade, Belgrade, Serbia; 114 VINCA Institute of Nuclear Sciences, University of Belgrade, Belgrade, Serbia; 115 Centro de Investigaciones Energéticas Medioambientales y Tecnológicas (CIEMAT), Madrid, Spain; 116 Universidad Autónoma de Madrid, Madrid, Spain; 117 Universidad de Oviedo, Instituto Universitario de Ciencias y Tecnologías Espaciales de Asturias (ICTEA), Oviedo, Spain; 118 Instituto de Física de Cantabria (IFCA), CSIC-Universidad de Cantabria, Santander, Spain; 119 University of Colombo, Colombo, Sri Lanka; 120 Department of Physics, University of Ruhuna, Matara, Sri Lanka; 121 CERN, European Organization for Nuclear Research, Geneva, Switzerland; 122 Paul Scherrer Institut, Villigen, Switzerland; 123 ETH Zurich-Institute for Particle Physics and Astrophysics (IPA), Zurich, Switzerland; 124 Universität Zürich, Zurich, Switzerland; 125 National Central University, Chung-Li, Taiwan; 126 National Taiwan University (NTU), Taipei, Taiwan; 127 High Energy Physics Research Unit, Department of Physics, Faculty of Science, Chulalongkorn University, Bangkok, Thailand; 128 Physics Department, Science and Art Faculty, Çukurova University, Adana, Turkey; 129 Middle East Technical University, Physics Department, Ankara, Turkey; 130 Bogazici University, Istanbul, Turkey; 131 Istanbul Technical University, Istanbul, Turkey; 132 Istanbul University, Istanbul, Turkey; 133 Yildiz Technical University, Istanbul, Turkey; 134 Institute for Scintillation Materials of National Academy of Science of Ukraine, Kharkiv, Ukraine; 135 National Science Centre, Kharkiv Institute of Physics and Technology, Kharkiv, Ukraine; 136 University of Bristol, Bristol, UK; 137 Rutherford Appleton Laboratory, Didcot, UK; 138 Imperial College, London, UK; 139 Brunel University, Uxbridge, UK; 140 Baylor University, Waco, TX USA; 141 Catholic University of America, Washington, DC USA; 142 The University of Alabama, Tuscaloosa, AL USA; 143 Boston University, Boston, MA USA; 144 Brown University, Providence, RI USA; 145 University of California, Davis, Davis, CA USA; 146 University of California, Los Angeles, CA USA; 147 University of California, Riverside, Riverside, CA USA; 148 University of California, San Diego, La Jolla, CA USA; 149 Department of Physics, University of California, Santa Barbara, Santa Barbara, CA USA; 150 California Institute of Technology, Pasadena, CA USA; 151 Carnegie Mellon University, Pittsburgh, PA USA; 152 University of Colorado Boulder, Boulder, CO USA; 153 Cornell University, Ithaca, NY USA; 154 Fermi National Accelerator Laboratory, Batavia, IL USA; 155 University of Florida, Gainesville, FL USA; 156 Florida State University, Tallahassee, FL USA; 157 Florida Institute of Technology, Melbourne, FL USA; 158 University of Illinois Chicago, Chicago, USA; 159 The University of Iowa, Iowa City, IA USA; 160 Johns Hopkins University, Baltimore, MD USA; 161 The University of Kansas, Lawrence, KS USA; 162 Kansas State University, Manhattan, KS USA; 163 University of Maryland, College Park, MD USA; 164 Massachusetts Institute of Technology, Cambridge, MA USA; 165 University of Minnesota, Minneapolis, MN USA; 166 University of Nebraska-Lincoln, Lincoln, NE USA; 167 State University of New York at Buffalo, Buffalo, NY USA; 168 Northeastern University, Boston, MA USA; 169 Northwestern University, Evanston, IL USA; 170 University of Notre Dame, Notre Dame, IN USA; 171 The Ohio State University, Columbus, OH USA; 172 Princeton University, Princeton, NJ USA; 173 University of Puerto Rico, Mayaguez, PR USA; 174 Purdue University, West Lafayette, IN USA; 175 Purdue University Northwest, Hammond, IN USA; 176 Rice University, Houston, TX USA; 177 University of Rochester, Rochester, NY USA; 178 Rutgers, The State University of New Jersey, Piscataway, NJ USA; 179 University of Tennessee, Knoxville, TN USA; 180 Texas A&M University, College Station, TX USA; 181 Texas Tech University, Lubbock, TX USA; 182 Vanderbilt University, Nashville, TN USA; 183 University of Virginia, Charlottesville, VA USA; 184 Wayne State University, Detroit, MI USA; 185 University of Wisconsin-Madison, Madison, WI USA; 186 Authors affiliated with an institute or an international laboratory covered by a cooperation agreement with CERN, Geneva, Switzerland; 187 Yerevan State University, Yerevan, Armenia; 188 TU Wien, Vienna, Austria; 189 Institute of Basic and Applied Sciences, Faculty of Engineering, Arab Academy for Science, Technology and Maritime Transport, Alexandria, Egypt; 190 Ghent University, Ghent, Belgium; 191 Universidade do Estado do Rio de Janeiro, Rio de Janeiro, Brazil; 192 Universidade Estadual de Campinas, Campinas, Brazil; 193 Federal University of Rio Grande do Sul, Porto Alegre, Brazil; 194 UFMS, Nova Andradina, Brazil; 195 Nanjing Normal University, Nanjing, China; 196 The University of Iowa, Iowa City, IA USA; 197 University of Chinese Academy of Sciences, Beijing, China; 198 China Center of Advanced Science and Technology, Beijing, China; 199 University of Chinese Academy of Sciences, Beijing, China; 200 China Spallation Neutron Source, Guangdong, China; 201 Henan Normal University, Xinxiang, China; 202 Université Libre de Bruxelles, Bruxelles, Belgium; 203 an institute or an international laboratory covered by a cooperation agreement with CERN, Geneva, Switzerland; 204 Suez University, Suez, Egypt; 205 British University in Egypt, Cairo, Egypt; 206 Purdue University, West Lafayette, IN USA; 207 Université de Haute Alsace, Mulhouse, France; 208 Istinye University, Istanbul, Turkey; 209 Tbilisi State University, Tbilisi, Georgia; 210 The University of the State of Amazonas, Manaus, Brazil; 211 University of Hamburg, Hamburg, Germany; 212 RWTH Aachen University, III. Physikalisches Institut A, Aachen, Germany; 213 Bergische University Wuppertal (BUW), Wuppertal, Germany; 214 Brandenburg University of Technology, Cottbus, Germany; 215 Forschungszentrum Jülich, Juelich, Germany; 216 CERN, European Organization for Nuclear Research, Geneva, Switzerland; 217 Institute of Nuclear Research ATOMKI, Debrecen, Hungary; 218 Universitatea Babes-Bolyai - Facultatea de Fizica, Cluj-Napoca, Romania; 219 MTA-ELTE Lendület CMS Particle and Nuclear Physics Group, Eötvös Loránd University, Budapest, Hungary; 220 HUN-REN Wigner Research Centre for Physics, Budapest, Hungary; 221 Physics Department, Faculty of Science, Assiut University, Assiut, Egypt; 222 Punjab Agricultural University, Ludhiana, India; 223 University of Visva-Bharati, Santiniketan, India; 224 Indian Institute of Science (IISc), Bangalore, India; 225 IIT Bhubaneswar, Bhubaneswar, India; 226 Institute of Physics, Bhubaneswar, India; 227 University of Hyderabad, Hyderabad, India; 228 Deutsches Elektronen-Synchrotron, Hamburg, Germany; 229 Isfahan University of Technology, Isfahan, Iran; 230 Sharif University of Technology, Tehran, Iran; 231 Department of Physics, University of Science and Technology of Mazandaran, Behshahr, Iran; 232 Department of Physics, Isfahan University of Technology, Isfahan, Iran; 233 Department of Physics, Faculty of Science, Arak University, Arak, Iran; 234 Helwan University, Cairo, Egypt; 235 Italian National Agency for New Technologies, Energy and Sustainable Economic Development, Bologna, Italy; 236 Centro Siciliano di Fisica Nucleare e di Struttura Della Materia, Catania, Italy; 237 Università degli Studi Guglielmo Marconi, Rome, Italy; 238 Scuola Superiore Meridionale, Università di Napoli ’Federico II’, Naples, Italy; 239 Fermi National Accelerator Laboratory, Batavia, IL USA; 240 Consiglio Nazionale delle Ricerche-Istituto Officina dei Materiali, Perugia, Italy; 241 Department of Applied Physics, Faculty of Science and Technology, Universiti Kebangsaan Malaysia, Bangi, Malaysia; 242 Consejo Nacional de Ciencia y Tecnología, Mexico City, Mexico; 243 Trincomalee Campus, Eastern University, Sri Lanka, Nilaveli, Sri Lanka; 244 Saegis Campus, Nugegoda, Sri Lanka; 245 National and Kapodistrian University of Athens, Athens, Greece; 246 Ecole Polytechnique Fédérale Lausanne, Lausanne, Switzerland; 247 Universität Zürich, Zurich, Switzerland; 248 Stefan Meyer Institute for Subatomic Physics, Vienna, Austria; 249 Laboratoire d’Annecy-le-Vieux de Physique des Particules, IN2P3-CNRS, Annecy-le-Vieux, France; 250 Near East University, Research Center of Experimental Health Science, Mersin, Turkey; 251 Konya Technical University, Konya, Turkey; 252 Izmir Bakircay University, Izmir, Turkey; 253 Adiyaman University, Adiyaman, Turkey; 254 Bozok Universitetesi Rektörlügü, Yozgat, Turkey; 255 Marmara University, Istanbul, Turkey; 256 Milli Savunma University, Istanbul, Turkey; 257 Kafkas University, Kars, Turkey; 258 Istanbul Okan University, Istanbul, Turkey; 259 Hacettepe University, Ankara, Turkey; 260 Erzincan Binali Yildirim University, Erzincan, Turkey; 261 Faculty of Engineering, Istanbul University-Cerrahpasa, Istanbul, Turkey; 262 Yildiz Technical University, Istanbul, Turkey; 263 Vrije Universiteit Brussel, Brussel, Belgium; 264 School of Physics and Astronomy, University of Southampton, Southampton, UK; 265 IPPP Durham University, Durham, UK; 266 Faculty of Science, Monash University, Clayton, Australia; 267 Università di Torino, Turin, Italy; 268 Bethel University, St. Paul, MN USA; 269 Karamanoğlu Mehmetbey University, Karaman, Turkey; 270 California Institute of Technology, Pasadena, CA USA; 271 United States Naval Academy, Annapolis, MD USA; 272 Ain Shams University, Cairo, Egypt; 273 Bingol University, Bingol, Turkey; 274 Georgian Technical University, Tbilisi, Georgia; 275 Sinop University, Sinop, Turkey; 276 Erciyes University, Kayseri, Turkey; 277 Horia Hulubei National Institute of Physics and Nuclear Engineering (IFIN-HH), Bucharest, Romania; 278 an institute or an international laboratory covered by a cooperation agreement with CERN, Geneva, Switzerland; 279 Texas A&M University at Qatar, Doha, Qatar; 280 Kyungpook National University, Daegu, Korea; 281 another institute or international laboratory covered by a cooperation agreement with CERN, Geneva, Switzerland; 282 Universiteit Antwerpen, Antwerpen, Belgium; 283 Yerevan Physics Institute, Yerevan, Armenia; 284 Northeastern University, Boston, MA USA; 285 Imperial College, London, UK; 286 Institute of Nuclear Physics of the Uzbekistan Academy of Sciences, Tashkent, Uzbekistan; 287 CERN, 1211 Geneva 23, Switzerland

## Abstract

A search is reported for charge-parity $CP$ violation in ${{{\textrm{D}}}^{{0}}} \rightarrow {{\textrm{K}} _{\text {S}}^{{0}}} {{\textrm{K}} _{\text {S}}^{{0}}}$ decays, using data collected in proton–proton collisions at $\sqrt{s} = 13\,\text {Te}\hspace{-.08em}\text {V}$ recorded by the CMS experiment in 2018. The analysis uses a dedicated data set that corresponds to an integrated luminosity of 41.6$\,\text {fb}^{-1}$, which consists of about 10 billion events containing a pair of b hadrons, nearly all of which decay to charm hadrons. The flavor of the neutral D meson is determined by the pion charge in the reconstructed decays ${{{\textrm{D}}}^{{*+}}} \rightarrow {{{\textrm{D}}}^{{0}}} {{{\mathrm{\uppi }}}^{{+}}}$ and ${{{\textrm{D}}}^{{*-}}} \rightarrow {\overline{{\textrm{D}}}^{{0}}} {{{\mathrm{\uppi }}}^{{-}}}$. The $CP$ asymmetry in ${{{\textrm{D}}}^{{0}}} \rightarrow {{\textrm{K}} _{\text {S}}^{{0}}} {{\textrm{K}} _{\text {S}}^{{0}}}$ is measured to be $A_{CP} ({{\textrm{K}} _{\text {S}}^{{0}}} {{\textrm{K}} _{\text {S}}^{{0}}} ) = (6.2 \pm 3.0 \pm 0.2 \pm 0.8)\%$, where the three uncertainties represent the statistical uncertainty, the systematic uncertainty, and the uncertainty in the measurement of the $CP$ asymmetry in the ${{{\textrm{D}}}^{{0}}} \rightarrow {{\textrm{K}} _{\text {S}}^{{0}}} {{{\mathrm{\uppi }}}^{{+}}} {{{\mathrm{\uppi }}}^{{-}}}$ decay. This is the first $CP$ asymmetry measurement by CMS in the charm sector as well as the first to utilize a fully hadronic final state.

## Introduction

The noninvariance of fundamental interactions under the combined charge-parity ($CP$) transformation is one of the necessary conditions for the generation of the observed baryon asymmetry in the universe [1]. In the standard model (SM), the $CP$ symmetry violation originates from a single phase in the Cabibbo–Kobayashi–Maskawa (CKM) quark mixing matrix [2, 3]. Extensive studies of $CP$ violation in weak interaction decays of strange and beauty mesons have been performed by many experiments, with all results to date being consistent with the predictions based on the CKM formalism [4]. However, the magnitude of $CP$ violation in the SM appears to be insufficient to explain the matter–antimatter asymmetry observed in the universe [5–7], suggesting the existence of sources of $CP$ violation beyond the SM. Charmed meson decays are the only meson decays involving an up-type quark where $CP$ violation can be studied, and are complementary to strange and beauty meson decays. In contrast to the K and B systems, $CP$ violation in charm mesons is severely suppressed by the Glashow–Iliopoulos–Maiani mechanism [8] and by the magnitude of the CKM elements [3]. Given the strong SM suppression, an observation of a significant $CP$ violation in D meson decays may indicate a contribution from new physics, which can be different from those relevant for down-type quark systems. The first observation of $CP$ violation in charm decays was recently reported by the LHCb Collaboration in a measurement of the $CP$ asymmetry ($A_{CP}$) difference between the ${{{\textrm{D}}}^{{0}}} \rightarrow {{{\textrm{K}}}^{{+}}} {{{\textrm{K}}}^{{-}}}$ and ${{{\textrm{D}}}^{{0}}} \rightarrow {{{\mathrm{\uppi }}}^{{+}}} {{{\mathrm{\uppi }}}^{{-}}}$ decays [9]. However, determining if this (or other) measurements of $CP$ violation is an indication of new physics is hampered by large theoretical uncertainties associated with long-distance contributions and nonperturbative effects [10].

**Fig. 1:**
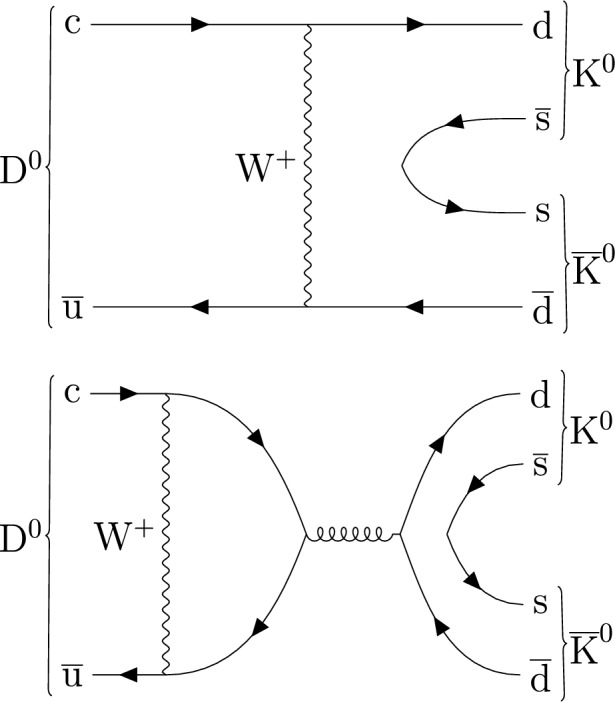
The decay of neutral charm meson to two neutral kaons: exchange (upper) and penguin annihilation (lower) diagrams

The ${{{\textrm{D}}}^{{0}}} \rightarrow {{\textrm{K}} _{\text {S}}^{{0}}} {{\textrm{K}} _{\text {S}}^{{0}}}$ decay proceeds through the W boson exchange and penguin annihilation Feynman diagrams, some examples of which are shown in Fig. 1, which results in a relatively small branching fraction of $( 1.41\pm 0.05) \times 10^{-4}$ [4]. In this figure and throughout this paper, charge-conjugate states are implied, unless otherwise indicated. Theoretical predictions indicate similar amplitudes and different phases for the two diagrams, which can result in $CP$ violation in this channel as large as a few percent [11–15] and therefore possibly within reach of current experiments.

The $CP$ asymmetry $A_{CP}$, for the ${{{\textrm{D}}}^{{0}}} \rightarrow {{\textrm{K}} _{\text {S}}^{{0}}} {{\textrm{K}} _{\text {S}}^{{0}}}$ decay, is defined as

1 $$\begin{aligned} A_{CP} ({{\textrm{K}} _{\text {S}}^{{0}}} {{\textrm{K}} _{\text {S}}^{{0}}} ) = \frac{\varGamma ({{{\textrm{D}}}^{{0}}} \rightarrow {{\textrm{K}} _{\text {S}}^{{0}}} {{\textrm{K}} _{\text {S}}^{{0}}} )-\varGamma ({\overline{{\textrm{D}}}^{{0}}} \rightarrow {{\textrm{K}} _{\text {S}}^{{0}}} {{\textrm{K}} _{\text {S}}^{{0}}} )}{\varGamma ({{{\textrm{D}}}^{{0}}} \rightarrow {{\textrm{K}} _{\text {S}}^{{0}}} {{\textrm{K}} _{\text {S}}^{{0}}} )+\varGamma ({\overline{{\textrm{D}}}^{{0}}} \rightarrow {{\textrm{K}} _{\text {S}}^{{0}}} {{\textrm{K}} _{\text {S}}^{{0}}} )}. \end{aligned}$$

The current world average for the time-integrated $CP$ asymmetry is $A_{CP} ({{\textrm{K}} _{\text {S}}^{{0}}} {{\textrm{K}} _{\text {S}}^{{0}}} ) =(-1.9 \pm 1.1){\%}$ [4], which is dominated by results from the LHCb [16] and Belle [17] Collaborations.

This paper presents the first $CP$ violation measurement by the CMS experiment in the charm sector. The flavor of the neutral D meson is determined from the pion charge found from reconstructing the decays ${{{\textrm{D}}}^{{*+}}} \rightarrow {{{\textrm{D}}}^{{0}}} {{{\mathrm{\uppi }}}^{{+}}}$ and ${{{\textrm{D}}}^{{*-}}} \rightarrow {\overline{{\textrm{D}}}^{{0}}} {{{\mathrm{\uppi }}}^{{-}}}$. We measure the $CP$ asymmetry difference, $\varDelta A_{CP}$, between the signal channel ${{{\textrm{D}}}^{{0}}} \rightarrow {{\textrm{K}} _{\text {S}}^{{0}}} {{\textrm{K}} _{\text {S}}^{{0}}}$ and the reference channel ${{{\textrm{D}}}^{{0}}} \rightarrow {{\textrm{K}} _{\text {S}}^{{0}}} {{{\mathrm{\uppi }}}^{{+}}} {{{\mathrm{\uppi }}}^{{-}}}$. The ${{{\textrm{D}}}^{{0}}} \rightarrow {{\textrm{K}} _{\text {S}}^{{0}}} {{{\mathrm{\uppi }}}^{{+}}} {{{\mathrm{\uppi }}}^{{-}}}$ $CP$ asymmetry has been previously measured [18] and found to be consistent with zero, as expected since this decay is not CKM-suppressed. Therefore, a significant deviation of $\varDelta A_{CP}$ from 0 would indicate $CP$ violation in the ${{{\textrm{D}}}^{{0}}} \rightarrow {{\textrm{K}} _{\text {S}}^{{0}}} {{\textrm{K}} _{\text {S}}^{{0}}}$ decay.

In proton–proton $(\textrm{pp})$ collision data, the number of ${{\textrm{D}}}^{{*+}}$ and ${{\textrm{D}}}^{{*-}}$ decays (signal events, *N*) are measured, where both ${{\textrm{D}}}^{{0}}$ and $\overline{{\textrm{D}}}^{{0}}$ are reconstructed in the ${{\textrm{K}} _{\text {S}}^{{0}}} {{\textrm{K}} _{\text {S}}^{{0}}}$ or ${{\textrm{K}} _{\text {S}}^{{0}}} {{{\mathrm{\uppi }}}^{{+}}} {{{\mathrm{\uppi }}}^{{-}}}$ decay modes. The “raw” asymmetry between these numbers, $A_{CP}^{\text {raw}}$ (defined in Eq. (2)), is different from the true asymmetry $A_{CP}$, due to the slightly different production cross sections $(\sigma )$ of ${{\textrm{D}}}^{{*+}}$ and ${{\textrm{D}}}^{{*-}}$ mesons, as well as to a possible difference in the detection efficiency ($\epsilon$) between ${{\textrm{D}}}^{{*+}}$ and ${{\textrm{D}}}^{{*-}}$. Because these three asymmetries are all small, the following relation can be written:

2 $$\begin{aligned} A_{CP}  &   \approx A_{CP}^{\text {raw}}-A_{CP}^{\text {pro}}-A_{CP}^{\text {det}},\quad \text {where} \nonumber \\ A_{CP}^{\text {raw}}  &   =\frac{N({{{\textrm{D}}}^{{*+}}} \rightarrow {{{\textrm{D}}}^{{0}}} {{{\mathrm{\uppi }}}^{{+}}} )-N({{{\textrm{D}}}^{{*-}}} \rightarrow {\overline{{\textrm{D}}}^{{0}}} {{{\mathrm{\uppi }}}^{{-}}} )}{N({{{\textrm{D}}}^{{*+}}} \rightarrow {{{\textrm{D}}}^{{0}}} {{{\mathrm{\uppi }}}^{{+}}} )+N({{{\textrm{D}}}^{{*-}}} \rightarrow {\overline{{\textrm{D}}}^{{0}}} {{{\mathrm{\uppi }}}^{{-}}} )}, \nonumber \\ A_{CP}^{\text {pro}}  &   =\frac{\sigma _{\textrm{pp}\rightarrow {{{\textrm{D}}}^{{*+}}} X}-\sigma _{\textrm{pp}\rightarrow {{{\textrm{D}}}^{{*-}}} X}}{\sigma _{\textrm{pp}\rightarrow {{{\textrm{D}}}^{{*+}}} X}+\sigma _{\textrm{pp}\rightarrow {{{\textrm{D}}}^{{*-}}} X}},\quad \text {and} \nonumber \\ A_{CP}^{\text {det}}  &   =\frac{\epsilon ({{{\textrm{D}}}^{{*+}}} \rightarrow {{{\textrm{D}}}^{{0}}} {{{\mathrm{\uppi }}}^{{+}}} )-\epsilon ({{{\textrm{D}}}^{{*-}}} \rightarrow {\overline{{\textrm{D}}}^{{0}}} {{{\mathrm{\uppi }}}^{{-}}} )}{\epsilon ({{{\textrm{D}}}^{{*+}}} \rightarrow {{{\textrm{D}}}^{{0}}} {{{\mathrm{\uppi }}}^{{+}}} )+\epsilon ({{{\textrm{D}}}^{{*-}}} \rightarrow {\overline{{\textrm{D}}}^{{0}}} {{{\mathrm{\uppi }}}^{{-}}} )}, \end{aligned}$$

where measuring the difference of $A_{CP}$ between the signal and reference channels, $A_{CP}^{\text {pro}}$ (${{\textrm{D}}}^{{*\pm }}$ production asymmetry) and $A_{CP}^{\text {det}}$ (${{\textrm{D}}}^{{*\pm }}$ detection asymmetry) cancel out, as they do not depend on the final state (${{\textrm{K}} _{\text {S}}^{{0}}} {{\textrm{K}} _{\text {S}}^{{0}}}$ or ${{\textrm{K}} _{\text {S}}^{{0}}} {{{\mathrm{\uppi }}}^{{+}}} {{{\mathrm{\uppi }}}^{{-}}}$):

3 $$\begin{aligned} \varDelta A_{CP}  &   \equiv A_{CP} ({{\textrm{K}} _{\text {S}}^{{0}}} {{\textrm{K}} _{\text {S}}^{{0}}} )- A_{CP} ({{\textrm{K}} _{\text {S}}^{{0}}} {{{\mathrm{\uppi }}}^{{+}}} {{{\mathrm{\uppi }}}^{{-}}} ) \nonumber \\  &   = A_{CP}^{\text {raw}} ({{\textrm{K}} _{\text {S}}^{{0}}} {{\textrm{K}} _{\text {S}}^{{0}}} ) - A_{CP}^{\text {raw}} ({{\textrm{K}} _{\text {S}}^{{0}}} {{{\mathrm{\uppi }}}^{{+}}} {{{\mathrm{\uppi }}}^{{-}}} ). \end{aligned}$$

The reference channel was chosen to be as similar as possible in kinematics, topology, and final-state signature to the signal channel, to ensure that the reconstruction efficiency asymmetries cancel in the measured difference of asymmetries, $\varDelta A_{CP}$.

The analysis uses proton–proton collisions data recorded by the CMS detector during the CERN LHC Run 2 in 2018, at $\sqrt{s}=13\,\text {Te}\hspace{-.08em}\text {V}$. It utilizes the B parking data set [19, 20], collected with a set of single-muon triggers with different minimum thresholds on the muon transverse momentum ($p_{\textrm{T}}$) and impact parameter with respect to the beamline. Different triggers were enabled depending on the instantaneous luminosity: as the luminosity decreased, less restrictive triggers were enabled, as allowed by the limited event rate to be processed by the data acquisition system and recorded on tape. The data set contains about $1.2\times 10^{10}$ events and corresponds to an integrated luminosity of $41.6{\,\text {fb}^{-1}}$. More details about this data set can be found in Ref. [19]. These triggers are intended to select events containing a semimuonic decay of a b hadron (or a semimuonic decay of c hadron that originated from a b-hadron decay). Since the trigger requires muons inconsistent with being produced in the primary interaction, most of such muons come from semileptonic decays of beauty hadrons, hence approximately 80% of the events in this sample include b hadrons [19, 20]. As beauty hadrons nearly always decay into charm hadrons, this data set also provides a rich sample of charm decays, making it suitable for $CP$ violation studies in the charm sector.

## The CMS detector

The central feature of the CMS apparatus is a superconducting solenoid of 6$\text { m}$ internal diameter, providing a magnetic field of 3.8$\text { T}$. Within the solenoid volume are a silicon pixel and strip tracker, a lead tungstate crystal electromagnetic calorimeter, and a brass and scintillator hadron calorimeter, each composed of a barrel and two endcap sections. Forward calorimeters extend the pseudorapidity ($\eta$) coverage provided by the barrel and endcap detectors. Muons are measured in gas-ionization detectors embedded in the steel flux-return yoke outside the solenoid. The reconstructed decays used by this analysis contain five pions in the final state. Pions are measured by the silicon tracker whose setup during the 2018 LHC running period, when the data used in this paper were recorded, consisted of 1856 silicon pixel [21] and 15 148 silicon strip detector modules. For non-isolated particles with $|\eta | < 3$ and $1< p_{\textrm{T}} < 10\,\text {Ge}\hspace{-.08em}\text {V}$, the track resolutions are typically 1.5% in $p_{\textrm{T}}$ and 20–75$\,\upmu \text {m}$ in the transverse impact parameter [22].

Events of interest are selected using a two-tiered trigger system. The first level, composed of custom hardware processors, uses information from the calorimeters and muon detectors to select events at a rate of around 100$\text { kHz}$ within a fixed latency of 4$\,\upmu \text {s}$  [23]. The second level, known as the high-level trigger, consists of a farm of processors running a version of the full event reconstruction software optimized for fast processing, and further reduces the event rate before data storage [24].

A more detailed description of the CMS detector, together with a definition of the coordinate system used and the relevant kinematic variables, can be found in Ref. [25].

## Simulated event samples

The simulated event samples used in this analysis are generated with pythia 8.230 [26]. The pythia output is interfaced with evtgen  [27] 1.3.0, which simulates various b and c hadron decays. The underlying event is also modeled with pythia using the CP5 [28] tune. Final-state photon radiation is modeled with photos 3.61 [29]. Samples with inclusive decays ${{{\textrm{B}}}^{{+}}} \rightarrow {{{\textrm{D}}}^{{*\pm }}} (\rightarrow {\textrm{D}} {{{\mathrm{\uppi }}}^{{\pm }}} )X$, ${{{\textrm{B}}}^{{0}}} \rightarrow {{{\textrm{D}}}^{{*\pm }}} (\rightarrow {\textrm{D}} {{{\mathrm{\uppi }}}^{{\pm }}} )X$, and prompt ${{{\textrm{D}}}^{{*\pm }}} \rightarrow {\textrm{D}} {{{\mathrm{\uppi }}}^{{\pm }}}$ were generated. The events were then passed through a detailed Geant4-based simulation [30] of the CMS detector, followed by the trigger and reconstruction algorithms identical to those used for the collision data.

## Reconstruction of charm meson decays

The reconstruction starts with finding ${{\textrm{K}} _{\text {S}}^{{0}}} \rightarrow {{{\mathrm{\uppi }}}^{{+}}} {{{\mathrm{\uppi }}}^{{-}}}$ candidates as described in Ref. [31]. The two oppositely-charged pion tracks are fit to a common vertex that is required to have a $\chi ^2$ fit probability $P_{\text {vtx}} > 1\%$. The dipion invariant mass must be within 20$\,\text {Me}\hspace{-.08em}\text {V}$ of the world average value of the ${\textrm{K}} _{\text {S}}^{{0}}$ meson mass [4], corresponding to approximately three times the mass resolution.

**Table 1:** Optimized selection criteria in the signal channel ${{\textrm{K}} _{\text {S}}^{{0}}} {{\textrm{K}} _{\text {S}}^{{0}}}$. The requirements on the ${\textrm{K}} _{\text {S}}^{{0}}$ candidates in the third and fourth lines are given first for the ${\textrm{K}} _{\text {S}}^{{0}}$ with larger $p_{\textrm{T}}$, then for the ${\textrm{K}} _{\text {S}}^{{0}}$ with lower $p_{\textrm{T}}$

Variable	Requirement
$|\eta |$ of the tagging pion from ${{{\textrm{D}}}^{{*\pm }}} \rightarrow {\textrm{D}} {{{\mathrm{\uppi }}}^{{\pm }}}$	<1.2
$p_{\textrm{T}}$ of the tagging pion from ${{{\textrm{D}}}^{{*\pm }}} \rightarrow {\textrm{D}} {{{\mathrm{\uppi }}}^{{\pm }}}$	>0.35$\,\text {Ge}\hspace{-.08em}\text {V}$
$p_{\textrm{T}} ({{\textrm{K}} _{\text {S}}^{{0}}})$	>2.2 and >1.0$\,\text {Ge}\hspace{-.08em}\text {V}$
${\textrm{K}} _{\text {S}}^{{0}}$ vertex displacement significance from the ${{\textrm{D}}}^{{0}}$ vertex in *xyz*	>7 and >9
${{\textrm{D}}}^{{0}}$ vertex displacement significance from the PV in *xy*	>2
${{\textrm{D}}}^{{0}}$ vertex displacement significance from the PV in *xyz*	>9
$P_{\text {vtx}} ({\textrm{D}} {{{\mathrm{\uppi }}}^{{\pm }}} )$	>5%
$P_{\text {vtx}} ({{\textrm{K}} _{\text {S}}^{{0}}} {{\textrm{K}} _{\text {S}}^{{0}}} )$	>1%
$P_{\text {vtx}} ({{{\mathrm{\uppi }}}^{{+}}} {{{\mathrm{\uppi }}}^{{-}}})$ for ${{\textrm{K}} _{\text {S}}^{{0}}} \rightarrow {{{\mathrm{\uppi }}}^{{+}}} {{{\mathrm{\uppi }}}^{{-}}}$	>1%
Angle between ${{\textrm{D}}}^{{0}}$ momentum and displacement from PV in *xyz*	<0.205$\text { rad}$
Angle between ${{\textrm{D}}}^{{0}}$ momentum and displacement from PV in *xy*	<0.237$\text { rad}$
Angle between ${{\textrm{D}}}^{{0}}$ momentum and displacement from beamline in *xy*	<0.237$\text { rad}$

In the signal channel, two ${{\textrm{K}} _{\text {S}}^{{0}}} \rightarrow {{{\mathrm{\uppi }}}^{{+}}} {{{\mathrm{\uppi }}}^{{-}}}$ candidates are each fit again with kinematic constraints to the ${\textrm{K}} _{\text {S}}^{{0}}$ mass, and subsequently, the ${\textrm{K}} _{\text {S}}^{{0}}$ candidates are fitted as two virtual tracks to a common vertex, assumed to be the ${{{\textrm{D}}}^{{0}}} \rightarrow {{\textrm{K}} _{\text {S}}^{{0}}} {{\textrm{K}} _{\text {S}}^{{0}}}$ decay vertex. The ${{\textrm{K}} _{\text {S}}^{{0}}} {{\textrm{K}} _{\text {S}}^{{0}}}$ invariant mass is required to be between 1.77 and 1.95$\,\text {Ge}\hspace{-.08em}\text {V}$, and the vertex fit probability must exceed 1%. Both ${\textrm{K}} _{\text {S}}^{{0}}$ decay vertices have to be displaced in three-dimensional (3D) space by at least one standard deviation (s.d.) from the fitted ${{\textrm{K}} _{\text {S}}^{{0}}} {{\textrm{K}} _{\text {S}}^{{0}}}$ vertex, and the corresponding pointing angle (the angle between the particle momentum and the vector joining the production vertex with its decay vertex) for each ${\textrm{K}} _{\text {S}}^{{0}}$ candidate is required to be less than $90^\circ$.

In the reference channel, after the single ${\textrm{K}} _{\text {S}}^{{0}}$ selection, two additional high-purity [32] and opposite-sign tracks with $p_{\textrm{T}} >0.6\,\text {Ge}\hspace{-.08em}\text {V}$ (and at least one of them with $p_{\textrm{T}} >0.7\,\text {Ge}\hspace{-.08em}\text {V}$) are selected. The ${{\textrm{K}} _{\text {S}}^{{0}}} {{{\mathrm{\uppi }}}^{{+}}} {{{\mathrm{\uppi }}}^{{-}}}$ combination is then fit to a common vertex, assumed to be the ${{\textrm{D}}}^{{0}}$ decay vertex, which must have a $P_{\text {vtx}} > 5\%$, and an invariant mass between 1.823 and 1.908$\,\text {Ge}\hspace{-.08em}\text {V}$, assuming charged-pion mass [4] for both tracks, which corresponds to approximately twice the mass resolution.

The primary vertex (PV) is selected from the reconstructed $\textrm{pp}$ interaction vertices as the one with the smallest pointing angle of the ${{\textrm{D}}}^{{0}}$ candidate. After the ${{\textrm{D}}}^{{0}}$ reconstruction, an additional track is added to form the ${{{\textrm{D}}}^{{*+}}} \rightarrow {{{\textrm{D}}}^{{0}}} {{{\mathrm{\uppi }}}^{{+}}}$ or ${{{\textrm{D}}}^{{*-}}} \rightarrow {\overline{{\textrm{D}}}^{{0}}} {{{\mathrm{\uppi }}}^{{-}}}$ candidates. A two-object vertex fit is performed to reconstruct the ${{\textrm{D}}}^{{*\pm }}$ decay vertex, which is required to have a $P_{\text {vtx}} > 1\%$. The ${{\textrm{D}}}^{{*+}}$ candidate invariant mass is determined from the refitted pion and ${{\textrm{D}}}^{{0}}$ four-momenta and then corrected by subtracting the difference between the reconstructed ${{\textrm{D}}}^{{0}}$ candidate mass and the world-average ${{\textrm{D}}}^{{0}}$ mass, to remove the effect of the ${{\textrm{D}}}^{{0}}$ detector mass resolution. The candidates are rejected if they are compatible with an incorrect decay topology that assumes negligible decay time of any of the ${\textrm{K}} _{\text {S}}^{{0}}$ candidates.

## Final selection criteria

A mixture of different triggers with varying thresholds in the data set makes it challenging to properly model the kinematic distributions of charm mesons in the simulation. Therefore, an optimization of the selection criteria is done using the experimental data directly. A two-dimensional (2D) fit to the distribution of $m({\textrm{D}} {{{\mathrm{\uppi }}}^{{\pm }}} )$ vs $m({{\textrm{K}} _{\text {S}}^{{0}}} {{\textrm{K}} _{\text {S}}^{{0}}} )$, similar to the one described below, is performed for the data (with the ${{\textrm{D}}}^{{*+}}$ and ${{\textrm{D}}}^{{*-}}$ samples merged) while the selection criteria are varied. The variables used in the optimization include the candidate $p_{\textrm{T}}$, $\eta$, $P_{\text {vtx}}$, distances between production and decay vertices for ${\textrm{K}} _{\text {S}}^{{0}}$ and ${{\textrm{D}}}^{{0}}$ candidates divided by their corresponding uncertainties, and corresponding pointing angles. The optimal criteria were chosen as those which result in the smallest relative uncertainty on the fitted signal yield. Cross-validation was used to ensure there is no bias due to statistical fluctuations in the data, via randomly splitting the data into six equal sub-samples, finding optimal criteria using five of them and applying them to the last part. The procedure is repeated six times (each time leaving out a different part of the full data set) and results in six almost identical sets of selection criteria. The average value for each selection is taken as the final selection criteria, presented in Table 1.

Similar selection criteria are applied to the reference channel, to minimize the differences in kinematic distributions between the signal and the reference channels: the only adjustment is that the scalar sum of the $p_{\textrm{T}}$ of the two pions that are not from the ${\textrm{K}} _{\text {S}}^{{0}}$ decay in the reference channel must exceed 1$\,\text {Ge}\hspace{-.08em}\text {V}$ and the single ${\textrm{K}} _{\text {S}}^{{0}}$ candidate in the reference channel must satisfy the requirements applied to the high-$p_{\textrm{T}}$ ${\textrm{K}} _{\text {S}}^{{0}}$ candidate in the signal channel.

## $A_{CP}$ measurement: reference channel

The signal and reference channels are found to have consistent $\eta$ and $\phi$ distributions, but slightly different $p_{\textrm{T}} ({{{\textrm{D}}}^{{*\pm }}})$ ones, and thus the detection and production asymmetries may not cancel out fully in the $\varDelta A_{CP}$ measurement. In order to suppress this effect, the reference channel data are reweighted to match the $p_{\textrm{T}} ({{{\textrm{D}}}^{{*\pm }}})$ distribution found in the signal channel, before splitting the samples by the pion charge.

**Fig. 2:**
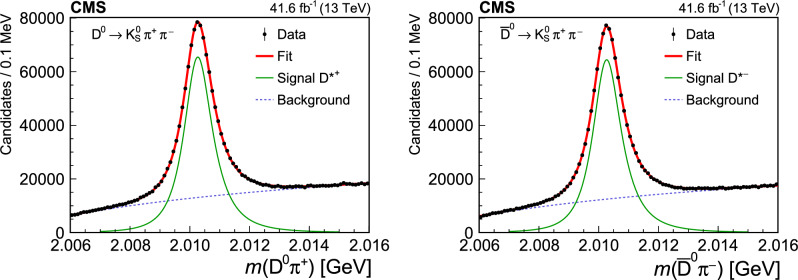
The ${{{\textrm{D}}}^{{0}}} {{{\mathrm{\uppi }}}^{{+}}}$ (left) and ${\overline{{\textrm{D}}}^{{0}}} {{{\mathrm{\uppi }}}^{{-}}}$ (right) invariant mass distributions for the ${{\textrm{K}} _{\text {S}}^{{0}}} {{{\mathrm{\uppi }}}^{{+}}} {{{\mathrm{\uppi }}}^{{-}}}$ channel, with the result of the fit to both distributions

To extract the raw $CP$ asymmetry, a simultaneous binned extended maximum likelihood fit is performed on the invariant mass distributions $m({\textrm{D}} {{{\mathrm{\uppi }}}^{{\pm }}} )$ of weighted ${{\textrm{D}}}^{{*+}}$ and ${{\textrm{D}}}^{{*-}}$ candidates. The signal in $x = m({\textrm{D}} {{{\mathrm{\uppi }}}^{{\pm }}} )$ is fitted with the $S_U$ Johnson transformation of the normal distribution [33], with the shape parameters shared between the ${{\textrm{D}}}^{{*+}}$ and ${{\textrm{D}}}^{{*-}}$ components while the signal yields are independent. The background is modeled with a modified threshold function $(x-x_0)^\alpha (1 + a x)$, where $x_0$ is the threshold value equal to the sum of the masses of ${{\textrm{D}}}^{{0}}$ and ${{\mathrm{\uppi }}}^{{+}}$, and $\alpha$ and *a* are floated in the fit and they are not shared between the ${{\textrm{D}}}^{{*+}}$ and the ${{\textrm{D}}}^{{*-}}$ background model. The results of the fit to the $m({\textrm{D}} {{{\mathrm{\uppi }}}^{{\pm }}} )$ distributions are presented in Fig. 2 and Table 2. The measured raw asymmetry is $A_{CP}^{\text {raw}} ({{\textrm{K}} _{\text {S}}^{{0}}} {{{\mathrm{\uppi }}}^{{+}}} {{{\mathrm{\uppi }}}^{{-}}} )= (0.78\pm 0.10)\%$, where the uncertainty is statistical only and accounts for the correlations found in the simultaneous fit.

## $A_{CP}$ measurement: signal channel

To reduce the statistical uncertainty arising from the signal channel yield, which is the dominant uncertainty in the analysis, the signal extraction is performed using a 2D unbinned maximum likelihood fit performed simultaneously on the ${{\textrm{D}}}^{{*+}}$ and ${{\textrm{D}}}^{{*-}}$ samples to the distribution of $m({\textrm{D}} {{{\mathrm{\uppi }}}^{{\pm }}} )$ *vs.* $m({{\textrm{K}} _{\text {S}}^{{0}}} {{\textrm{K}} _{\text {S}}^{{0}}} )$. The fit function consists of the following components:

- ${{{\textrm{D}}}^{{0}}} \,\times \,{{{\textrm{D}}}^{{*+}}}$, the signal component;
- ${{{\textrm{D}}}^{{0}}} \,\times \,\, bkg$, for events containing genuine ${{\textrm{D}}}^{{0}}$ and background pion combinations;
- $bkg\,\,\times \,\,bkg$, for the background in both dimensions,

where each component is a product of two one-dimensional (1D) functions. For the ${{\textrm{D}}}^{{*\pm }}$ signal, the Johnson function is used with all signal shape parameters fixed to those found in the fit to the reference channel. This approach is verified to be reasonable using simulated event samples. The ${{\textrm{D}}}^{{0}}$ signal is modeled with a sum of two Johnson functions, all parameters of which are fixed to values determined from the simulation, except for a single free parameter that is used to scale the width. The background in $x = m({\textrm{D}} {{{\mathrm{\uppi }}}^{{\pm }}} )$ is modeled with the same function as in the reference channel. The background in $y = m({{\textrm{K}} _{\text {S}}^{{0}}} {{\textrm{K}} _{\text {S}}^{{0}}} )$ is described with an exponential function $\exp (\beta y)$, where $\beta$ is floating in the fit, plus a Gaussian function with free parameters to describe the partially-reconstructed background from the ${{\textrm{D}} _{\text {s}}^{{\pm }}} \rightarrow {{\textrm{K}} _{\text {S}}^{{0}}} {{\textrm{K}} _{\text {S}}^{{0}}} {{{\mathrm{\uppi }}}^{{\pm }}}$ decay producing an excess at about 1.83$\,\text {Ge}\hspace{-.08em}\text {V}$ in the ${{\textrm{K}} _{\text {S}}^{{0}}} {{\textrm{K}} _{\text {S}}^{{0}}}$ invariant mass distribution.

**Fig. 3:**
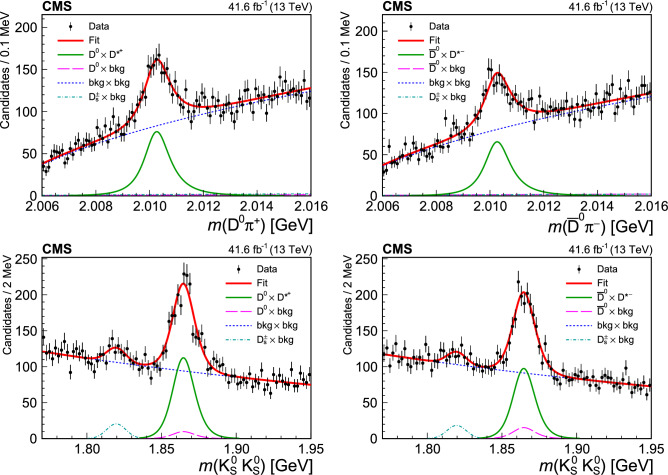
The invariant mass distributions for ${{\textrm{D}}}^{{*+}}$ candidates (left) and ${{\textrm{D}}}^{{*-}}$ candidates (right), with the $m({\textrm{D}} {{{\mathrm{\uppi }}}^{{\pm }}} )$ distributions in the upper row and the $m({{\textrm{K}} _{\text {S}}^{{0}}} {{\textrm{K}} _{\text {S}}^{{0}}} )$ distributions in the lower row. Projections of the simultaneous 2D fit are also shown

The projections of the data and the 2D fit on both axes are shown in Fig. 3; additional projections in sub-ranges are shown in Appendix A. The fit results are listed in Table 3. The measured raw asymmetry is $A_{CP}^{\text {raw}} ({{\textrm{K}} _{\text {S}}^{{0}}} {{\textrm{K}} _{\text {S}}^{{0}}} )= (7.1 \pm 3.0)\%$ and in combination with the results of Sect. 6 the $A_{CP}$ difference is measured to be $\varDelta A_{CP} = (6.3 \pm 3.0)\%$, where the uncertainty is statistical only and accounts for the correlations found in the simultaneous fit.

**Table 2:** Results of the fit to the selected ${{{\textrm{D}}}^{{*+}}} \rightarrow {{{\textrm{D}}}^{{0}}} {{{\mathrm{\uppi }}}^{{+}}}$ and ${{{\textrm{D}}}^{{*-}}} \rightarrow {\overline{{\textrm{D}}}^{{0}}} {{{\mathrm{\uppi }}}^{{-}}}$ candidates, where ${{{\textrm{D}}}^{{0}}} \,({\overline{{\textrm{D}}}^{{0}}}) \rightarrow {{\textrm{K}} _{\text {S}}^{{0}}} {{{\mathrm{\uppi }}}^{{+}}} {{{\mathrm{\uppi }}}^{{-}}}$. The ${{\textrm{D}}}^{{*\pm }}$ signal yields *N* given in the second column are used in the evaluation of $A_{CP}^{\text {raw}}$. The uncertainties are statistical only

Decay	*N*	$\chi ^2$ with 100 bins
${{{\textrm{D}}}^{{*+}}} \rightarrow {{{\textrm{D}}}^{{0}}} {{{\mathrm{\uppi }}}^{{+}}}$	$944\,800\pm 3\,500$	78
${{{\textrm{D}}}^{{*-}}} \rightarrow {\overline{{\textrm{D}}}^{{0}}} {{{\mathrm{\uppi }}}^{{-}}}$	$930\,150\pm 3\,400$	93

## Systematic uncertainties

The measured difference in the asymmetries is largely insensitive to many systematic uncertainties that would affect a measurement of $A_{CP}$ in a single channel, such as the difficult-to-measure production and detection asymmetries that would need a dedicated calibration procedure.

Uncertainties related to the choice of the signal and background models are calculated separately using alternative models and assessing the observed variations in $\varDelta A_{CP}$.

In the baseline approach, the signal in the $m({\textrm{D}} {{{\mathrm{\uppi }}}^{{\pm }}} )$ invariant mass distribution is modeled with the Johnson function [33]. As an alternative, we use a Johnson+Gaussian function with a common mean. Another alternative is a sum of two Crystal Ball functions [34]. For each case, the reference channel is fit as a first step, then the obtained shape parameters are fixed in the 2D fit to the signal channel. Other components of the 2D fit remain unchanged from the baseline fit. The largest deviation in $\varDelta A_{CP}$ from the baseline value is taken as a systematic uncertainty.

The baseline signal function for the $m({{\textrm{K}} _{\text {S}}^{{0}}} {{\textrm{K}} _{\text {S}}^{{0}}} )$ invariant mass distribution is a sum of two Johnson functions. As an alternative, we use a Johnson+Gaussian function or a sum of two Crystal Ball functions. These variations have no effect on the fit of the reference channel, just on that of the signal channel. The largest deviation in $\varDelta A_{CP}$ from the baseline value is taken as a systematic uncertainty.

**Table 3:** Results of the 2D fit to the selected ${{{\textrm{D}}}^{{*+}}} \rightarrow {{{\textrm{D}}}^{{0}}} {{{\mathrm{\uppi }}}^{{+}}}$ and ${{{\textrm{D}}}^{{*-}}} \rightarrow {\overline{{\textrm{D}}}^{{0}}} {{{\mathrm{\uppi }}}^{{-}}}$ candidates, where ${{{\textrm{D}}}^{{0}}} \,({\overline{{\textrm{D}}}^{{0}}}) \rightarrow {{\textrm{K}} _{\text {S}}^{{0}}} {{\textrm{K}} _{\text {S}}^{{0}}}$. The ${{\textrm{D}}}^{{*\pm }}$ signal yields *N* given in the second column are used in the evaluation of $A_{CP}^{\text {raw}}$. The $\chi ^2$ corresponds to the fit projection with 100 bins in the $x = m({\textrm{D}} {{{\mathrm{\uppi }}}^{{\pm }}} )$ axis and 90 bins in the $y = m({{\textrm{K}} _{\text {S}}^{{0}}} {{\textrm{K}} _{\text {S}}^{{0}}} )$ axis, as shown in Fig 3. The uncertainties are statistical only

Decay	*N*	$\chi ^2$ (*x* axis)	$\chi ^2$ (*y* axis)
${{{\textrm{D}}}^{{*+}}} \rightarrow {{{\textrm{D}}}^{{0}}} {{{\mathrm{\uppi }}}^{{+}}}$	$1 095 \pm 46$	77	90
${{{\textrm{D}}}^{{*-}}} \rightarrow {\overline{{\textrm{D}}}^{{0}}} {{{\mathrm{\uppi }}}^{{-}}}$	$\phantom {0}951\pm 44$	93	62

The baseline background model in the $x = m({\textrm{D}} {{{\mathrm{\uppi }}}^{{\pm }}} )$ distribution is $(x-x_0)^\alpha (1 + a x)$. An alternative background model is obtained by changing the function multiplying the threshold function from a linear polynomial to an exponential function. The baseline background model in the $m({{\textrm{K}} _{\text {S}}^{{0}}} {{\textrm{K}} _{\text {S}}^{{0}}} )$ distribution is an exponential function and an exponential multiplied by a linear polynomial is used as an alternative. These variations are taken as independent systematic uncertainties.

In the signal channel fit, there is a contribution from the ${{\textrm{D}} _{\text {s}}^{{\pm }}} \rightarrow {{\textrm{K}} _{\text {S}}^{{0}}} {{\textrm{K}} _{\text {S}}^{{0}}} {{{\mathrm{\uppi }}}^{{\pm }}}$ decay, which is modeled by a Gaussian with free parameters. As an alternative, we remove this reflection by restricting the fit range to be $m({{\textrm{K}} _{\text {S}}^{{0}}} {{\textrm{K}} _{\text {S}}^{{0}}} ) >1.835\,\text {Ge}\hspace{-.08em}\text {V}$, and the deviation from the baseline is included as a systematic uncertainty.

To assess the systematic uncertainty related to the $p_{\textrm{T}}$ reweighting, we vary the parameters of the reweighting function within their uncertainties. As an alternative, we consider the weights depending on the $p_{\textrm{T}}$ of the low-momentum pion that is used for the flavor tagging instead of $p_{\textrm{T}} ({{{\textrm{D}}}^{{*\pm }}})$. The largest change is taken as a systematic uncertainty related to the reweighting.

Differences in $A_{CP}^{\text {pro}}$ and $A_{CP}^{\text {det}}$ between the two channels are expected to be reproduced by the simulation of the processes and the detector. Checking the reweighted reference channel and signal channel in simulation show that the $p_{\textrm{T}}$-, $\eta$-, and $\phi$-dependent asymmetries are consistent with zero as is the integrated value of $(-0.13\pm 0.34)\%$. Therefore, no systematic uncertainty is assessed.

If multiple candidates in the same event are removed by keeping only the one with the highest ${{\textrm{D}}}^{{*\pm }}$ vertex fit probability, the resulting $\varDelta A_{CP}$ changes negligibly and no corresponding systematic uncertainty is assigned. Pion charge misidentification was shown to have a negligible effect as well.

All systematic uncertainties described above are uncorrelated and summarized in Table 4 together with the total systematic uncertainty, calculated as the sum in quadrature of the different contributions.

**Table 4:** Absolute systematic uncertainties in the measurement of $\varDelta A_{CP}$

Source	Uncertainty (%)
$m({\textrm{D}} {{{\mathrm{\uppi }}}^{{\pm }}} )$ signal model	0.10
$m({\textrm{D}} {{{\mathrm{\uppi }}}^{{\pm }}} )$ background model	0.02
$m({{\textrm{K}} _{\text {S}}^{{0}}} {{\textrm{K}} _{\text {S}}^{{0}}} )$ signal model	0.04
$m({{\textrm{K}} _{\text {S}}^{{0}}} {{\textrm{K}} _{\text {S}}^{{0}}} )$ background model	0.02
$m({{\textrm{K}} _{\text {S}}^{{0}}} {{\textrm{K}} _{\text {S}}^{{0}}} )$ fit range	0.06
Reweighting	0.09
Total	0.16

**Fig. 4:**
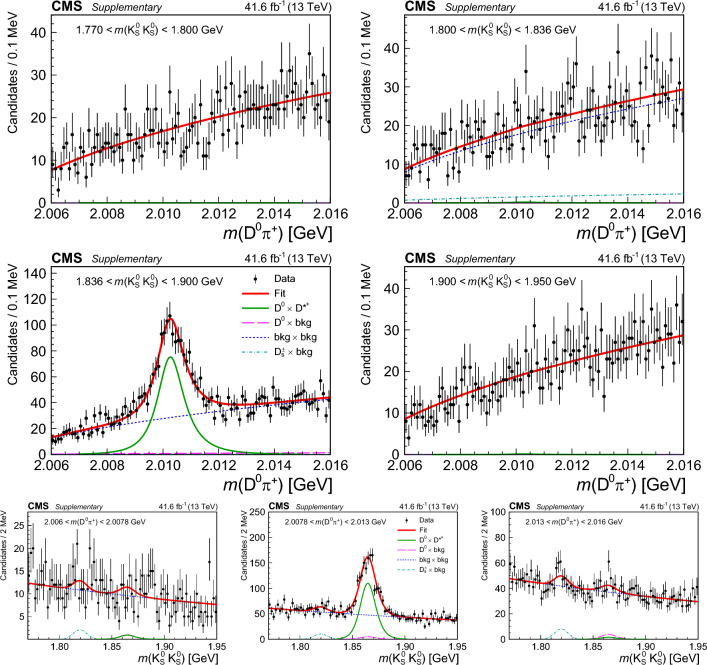
Results of the 2D fit to the $m({\textrm{D}} {{{\mathrm{\uppi }}}^{{\pm }}} ) \times m({{\textrm{K}} _{\text {S}}^{{0}}} {{\textrm{K}} _{\text {S}}^{{0}}} )$ for the signal channel, ${{\textrm{D}}}^{{*+}}$ candidates. Upper and middle rows show 1D projections of the 2D fit on $m({{{\textrm{D}}}^{{0}}} {{{\mathrm{\uppi }}}^{{+}}} )$ in ranges of $m({{\textrm{K}} _{\text {S}}^{{0}}} {{\textrm{K}} _{\text {S}}^{{0}}} )$: left sideband (upper left), region of ${{\textrm{D}} _{\text {s}}^{{\pm }}} \rightarrow {{\textrm{K}} _{\text {S}}^{{0}}} {{\textrm{K}} _{\text {S}}^{{0}}} {{{\mathrm{\uppi }}}^{{\pm }}}$ contamination (upper right), signal region of ${{\textrm{K}} _{\text {S}}^{{0}}} {{\textrm{K}} _{\text {S}}^{{0}}}$ (middle left), and right sideband (middle right). Lower row shows 1D projections of the 2D fit on $m({{\textrm{K}} _{\text {S}}^{{0}}} {{\textrm{K}} _{\text {S}}^{{0}}} )$ in ranges of $m({{{\textrm{D}}}^{{0}}} {{{\mathrm{\uppi }}}^{{+}}} )$: left sideband (left), signal region of ${{{\textrm{D}}}^{{0}}} {{{\mathrm{\uppi }}}^{{+}}}$ (center), and right sideband (right)

**Fig. 5:**
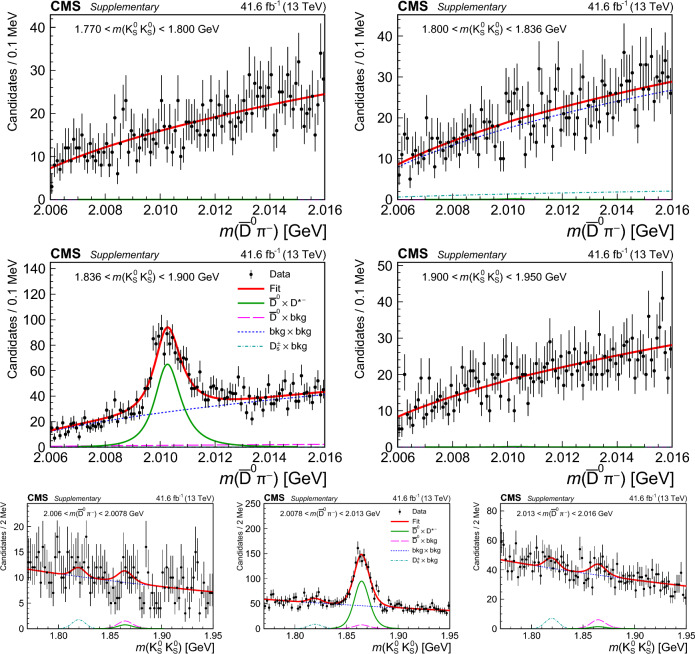
Results of the 2D fit to the $m({\textrm{D}} {{{\mathrm{\uppi }}}^{{\pm }}} ) \times m({{\textrm{K}} _{\text {S}}^{{0}}} {{\textrm{K}} _{\text {S}}^{{0}}} )$ for the signal channel, ${{\textrm{D}}}^{{*-}}$ candidates. Upper and middle rows show 1D projections of the 2D fit on $m({\overline{{\textrm{D}}}^{{0}}} {{{\mathrm{\uppi }}}^{{-}}} )$ in ranges of $m({{\textrm{K}} _{\text {S}}^{{0}}} {{\textrm{K}} _{\text {S}}^{{0}}} )$: left sideband (upper left), region of ${{\textrm{D}} _{\text {s}}^{{\pm }}} \rightarrow {{\textrm{K}} _{\text {S}}^{{0}}} {{\textrm{K}} _{\text {S}}^{{0}}} {{{\mathrm{\uppi }}}^{{\pm }}}$ contamination (upper right), signal region of ${{\textrm{K}} _{\text {S}}^{{0}}} {{\textrm{K}} _{\text {S}}^{{0}}}$ (middle left), and right sideband (middle right). Lower row shows 1D projections of the 2D fit on $m({{\textrm{K}} _{\text {S}}^{{0}}} {{\textrm{K}} _{\text {S}}^{{0}}} )$ in ranges of $m({\overline{{\textrm{D}}}^{{0}}} {{{\mathrm{\uppi }}}^{{-}}} )$: left sideband (left), signal region of ${\overline{{\textrm{D}}}^{{0}}} {{{\mathrm{\uppi }}}^{{-}}}$ (center), and right sideband (right)

## Summary

A measurement of $CP$ violation in ${{\textrm{D}}}^{{0}}$ decays is reported, using proton–proton collision data collected at $\sqrt{s} = 13\,\text {Te}\hspace{-.08em}\text {V}$ with a novel high-rate data stream (B parking). These data correspond to an integrated luminosity of 41.6$\,\text {fb}^{-1}$ and include about 10 billion events containing beauty hadron decays. The difference in the $CP$ asymmetries between ${{{\textrm{D}}}^{{0}}} \rightarrow {{\textrm{K}} _{\text {S}}^{{0}}} {{\textrm{K}} _{\text {S}}^{{0}}}$ and ${{{\textrm{D}}}^{{0}}} \rightarrow {{\textrm{K}} _{\text {S}}^{{0}}} {{{\mathrm{\uppi }}}^{{+}}} {{{\mathrm{\uppi }}}^{{-}}}$ is measured to be:

4 $$\begin{aligned} \begin{aligned} \varDelta A_{CP}&\equiv A_{CP} ({{\textrm{K}} _{\text {S}}^{{0}}} {{\textrm{K}} _{\text {S}}^{{0}}} )- A_{CP} ({{\textrm{K}} _{\text {S}}^{{0}}} {{{\mathrm{\uppi }}}^{{+}}} {{{\mathrm{\uppi }}}^{{-}}} ) \\&= \left( 6.3 \pm 3.0\,\text {(stat)} \pm 0.2\,\text {(syst)} \right) \%. \end{aligned} \end{aligned}$$

Using the world-average value of $A_{CP} ({{\textrm{K}} _{\text {S}}^{{0}}} {{{\mathrm{\uppi }}}^{{+}}} {{{\mathrm{\uppi }}}^{{-}}} ) = (-0.1 \pm 0.8)\%$ [4, 18, 35], we report the measurement

5 $$\begin{aligned} A_{CP} ({{\textrm{K}} _{\text {S}}^{{0}}} {{\textrm{K}} _{\text {S}}^{{0}}} ) = (6.2 \pm 3.0 \pm 0.2 \pm 0.8)\%, \end{aligned}$$

where the three uncertainties represent the statistical uncertainty, the systematic uncertainty, and the uncertainty in the measurement of the $CP$ asymmetry in the ${{{\textrm{D}}}^{{0}}} \rightarrow {{\textrm{K}} _{\text {S}}^{{0}}} {{{\mathrm{\uppi }}}^{{+}}} {{{\mathrm{\uppi }}}^{{-}}}$ decay. The measured value is consistent with no $CP$ violation within 2.0 standard deviations. Likewise, it is consistent with the LHCb [16] and the Belle measurements [17] at the level of 2.7 and 1.8 standard deviations, respectively. Tabulated results are provided in the HEPData record for this analysis [36]. This is the first CMS search for $CP$ violation in the charm sector, paving the way for future measurements with more data, using new techniques, and in other channels.

## Data Availability

This manuscript has no associated data. [Authors’ comment: Release and preservation of data used by the CMS Collaboration as the basis for publications is guided by the CMS data preservation, re-use, and open access policy.]

## Appendix A: Additional projections of the 2D fit

Figures 4 and 5 show the projections of the 2D fit in the signal channel in subranges of the mass variables. The top and middle rows show 1D projections of the 2D fit on $m({\textrm{D}} {{{\mathrm{\uppi }}}^{{\pm }}} )$ in ranges of $m({{\textrm{K}} _{\text {S}}^{{0}}} {{\textrm{K}} _{\text {S}}^{{0}}} )$: left sideband, region of ${{\textrm{D}} _{\text {s}}^{{\pm }}} \rightarrow {{\textrm{K}} _{\text {S}}^{{0}}} {{\textrm{K}} _{\text {S}}^{{0}}} {{{\mathrm{\uppi }}}^{{\pm }}}$ contamination, signal region of ${{\textrm{K}} _{\text {S}}^{{0}}} {{\textrm{K}} _{\text {S}}^{{0}}}$, and right sideband. The lower three plots show 1D projections of the 2D fit on $m({{\textrm{K}} _{\text {S}}^{{0}}} {{\textrm{K}} _{\text {S}}^{{0}}} )$ in ranges of $m({\textrm{D}} {{{\mathrm{\uppi }}}^{{\pm }}} )$: left sideband, signal region of ${\textrm{D}} {{{\mathrm{\uppi }}}^{{\pm }}}$, and right sideband.

## Code Availability Statement

The CMS core software is publicly available on GitHub (https://github.com/cms-sw/cmssw).
